# A critique of recent economic evaluations of community water fluoridation

**DOI:** 10.1179/2049396714Y.0000000093

**Published:** 2015-03

**Authors:** Lee Ko, Kathleen M Thiessen

**Affiliations:** 1 Oakland, CA, USA; 2 Oak Ridge Center for Risk Analysis, Oak Ridge, TN, USA

**Keywords:** Water fluoridation, Economic evaluation, Cost of water fluoridation, Caries prevention, Cost benefit, Cost effectiveness, Effectiveness in adults, Dental fluorosis

## Abstract

**Background:**

Although community water fluoridation (CWF) results in a range of potential contaminant exposures, little attention has been given to many of the possible impacts. A central argument for CWF is its cost-effectiveness. The U.S. Government states that $1 spent on CWF saves $38 in dental treatment costs.

**Objective:**

To examine the reported cost-effectiveness of CWF.

**Methods:**

Methods and underlying data from the primary U.S. economic evaluation of CWF are analyzed and corrected calculations are described. Other recent economic evaluations are also examined.

**Results:**

Recent economic evaluations of CWF contain defective estimations of both costs and benefits. Incorrect handling of dental treatment costs and flawed estimates of effectiveness lead to overestimated benefits. The real-world costs to water treatment plants and communities are not reflected.

**Conclusions:** Minimal correction reduced the savings to $3 per person per year (PPPY) for a best-case scenario, but this savings is eliminated by the estimated cost of treating dental fluorosis.

## INTRODUCTION

The USA and several other countries practice community water fluoridation (CWF), which has been promoted as the preferred solution to reduce caries for over half a century.^1^ Approximately two-thirds of the U.S. population is treated in this manner according to the Centers for Disease Control and Prevention (CDC).^2^ Community water fluoridation programs have increased water fluoride concentrations to 0.7–1.2 mg/l [0.7–1.2 parts per million (ppm)], although a 2011 proposed recommendation, if finalized, would decrease this to 0.7 mg/l.^3^

Community water fluoridation is a unique delivery mode of public health care in that fluoride is administered to everyone who drinks the water, regardless of dental status or needs, and at an amount proportional to the water consumed from the fluoridated source, which can range from zero to several liters per day.^4^ At the same time, because most community water is not consumed by people, CWF results in dispersion of a regulated contaminant, fluoride, to the greater environment via wastewater treatment plants, storm sewer systems, and use on lawns and gardens. Fluoridation chemicals typically contain other regulated contaminants (e.g. arsenic), extending the possibility of human exposures and environmental dispersal.^5^^–^8

A central argument for using CWF to reduce tooth decay is the cost savings claimed by the CDC:^9^ Every $1 invested in this preventive measure yields approximately $38 savings in dental treatment costs. This argument is repeated by the majority of state governments (Appendix 1) and is frequently cited by proponents to argue for initiating or maintaining CWF.

All $ signs in this paper refer to US$ unless otherwise indicated. However, statements such as $1 saves $38 are currency neutral.

The CDC’s estimate is calculated from the per person per year (PPPY) savings reported by Griffin *et al.*:^10^ With base-case assumptions, the annual per person cost savings resulting from fluoridation ranged from $15.95 in very small communities to $18.62 in large communities.^10^^i^ Table 1 summarizes Griffin *et al.*’s results by population size. The CDC derived the $1-saves-$38 claim by scaling the $0.50 cost and the $18.62 savings estimate for large systems (>20,000 people) to get $0.50 : $18.62 ≈ $1 : $38. However, this derivation is not valid because it implies scalability where scalability does not apply: spending more on CWF does not increase caries aversion or caries to be averted.

**Table 1:** Estimated costs and savings of community water fluoridation (CWF) for communities of various sizes from Griffin *et al.*^10^

Population size	Estimated cost* ($, PPPY)^‡^	Estimated savings*^†^ ($, PPPY)^‡^
<5,000	3.17	15.95
5,000–9,999	1.64	17.48
10,000–20,000	1.06	18.06
>20,000	0.50	18.62

*Based on a 4% discount rate.

^†^Calculated with the base-case gross savings of $19.12.

^‡^Per person per year.

Griffin *et al.*^10^ is the prime example of a body of work that attempts to evaluate the economics of CWF. As the most comprehensive and most cited work, it will be our focus. We limit our analysis to the smallest and largest systems in keeping with the CDC’s report.^9^ We also examined and comment on additional CWF cost-benefit analyses (Appendix 2).^ii^

### Key steps in Griffin *et al.*^10^

A 1989 workshop^12^ at the University of Michigan discussed the cost-effectiveness of CWF and other caries prevention programs, with cost estimates based primarily on data from Garcia.^13^ A 1992 paper by Ringelberg *et al.*^14^ improved upon Garcia’s cost estimates, and Griffin *et al.*^10^ produced their cost estimates (Table 1) by applying minor adjustments to the results of Ringelberg *et al.*,^14^ as described later in this paper.

Griffin *et al.*^10^ adopted a “societal perspective” and defined benefit as the cost of averted dental fees and associated productivity losses. They used a 4% discount rate for the main result of $19.12 gross savings. Griffin *et al.*’s stated assumptions and key intermediate results, organized into a set of key inputs, are provided in Table 2. Note that Input (c) differs from Assumption (3) in the timing of treatment — the authors’ calculation was consistent with treatment in the same year. The following steps explain how Griffin *et al.* obtained their value:

**Table 2:** Stated assumptions of Griffin *et al.*^10^ and key inputs of the calculation of benefits

Stated assumptions	Key inputs
(1) The benefit is decay prevented and begins at age 6 years	(a) Benefit is the number of decayed tooth surfaces that would otherwise have been treated
(2) The benefit is constant over time(3) All decay is eventually treated(4) The adverse effects are negligible(5) Dental fees equal the cost of dental resources(6) A decayed tooth surface will always receive a one-surface restoration	(b) Benefit in dollar amounts, or gross savings, is quantified in terms of averted dental fees for amalgam fillings and averted productivity losses due to a visit to a restorative dentist(c) Every decayed surface results in a 1-hour dental visit for a single-surface restoration in the same year it occurs(d) The dental fee for a single-surface filling is $54, and the productivity loss from the visit is $18 (the U.S. average hourly wage)(e) A single-surface filling is replaced every 12 years with another single-surface filling, up to age 65 years(f) It takes one year of exposure for CWF to begin to prevent tooth decay(g) CWF averts 0.19 decayed tooth surfaces per person per year (PPPY) on average
	(h) The same rate of caries aversion applies from age 6 to 64 years

CWF: community water fluoridation.

Step 1: From Input (d), restoring one decayed tooth surface costs $54+$18 = $72.
Step 2: As described by Input (e) the lifetime costs of a decayed surface include future replacement fillings; the number of replacements depends on when the decay occurs. Future replacement costs are discounted to arrive at a present value. The first avertable filling is discounted 1 year because of Input (f) in Table 2; replacements take place every 12 years up to age 65 years, based on Input (e).iii For example, for a child age 12.5 years, the lifetime cost at a 4% discount rate was estimated to be $159.61 as shown in the following equation:
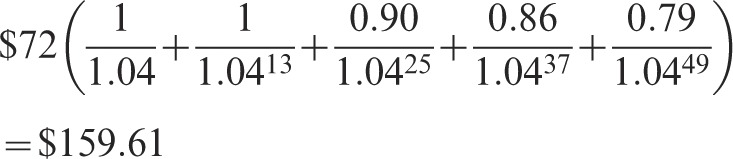
(1)Thus the last replacement takes place at age 12.5+1+(4×12) = 61.5 in this example. The cost for each filling or replacement filling is adjusted by a factor (the numerator of the term) representing the probability that the tooth remains at the replacement age.
Step 3: Calculate the national average, using the population distribution in Table 3. Use the midpoint to represent the group in each bracket, e.g. Equation (1) gives the lifetime benefit of an averted decay surface for the 6–19 age group, based on the midpoint, age 12.5 years.
Step 4: The calculation is repeated for each age bracket, except the first and the last age brackets as described by Input (h) in Table 2. Summing the weighted costs gives $100.62 as the national average lifetime cost averted per decayed surface.
Step 5: As CWF averts 0.19 decayed surfaces PPPY, as described by Input (g), the benefit of CWF is thus

**Table 3:** Griffin *et al.*^10^ weighted per person discounted lifetime cost of carious surface initially occurring at various ages

Age (years)	Discounted expected lifetime cost of decayed surface ($)	1996 U.S. population (%)	Weighted cost ($)
0–5		8.4	
6–19	159.61	20.4	32.56
20–24	146.95	6.8	9.99
25–29	144.86	7.2	10.43
30–34	128.24	8.3	10.64
35–39	127.76	8.5	10.86
40–44	105.12	7.7	8.09
45–49	105.55	6.6	6.97
50–54	106.42	5.2	5.53
55–59	69.23	4.2	2.91
60–64	69.23	3.8	2.63
65+		12.8	
Total		100	100.62

Gross saving = $100.62×0.19 = $19.12 PPPY

## COSTS

Griffin *et al.*^10^ based their cost estimates for CWF on Ringelberg *et al.*,^14^ except that the numbers were adjusted to 1995 dollars, and a different grouping of community sizes was used. Griffin *et al.* devote one paragraph to their cost estimates.^10^

### Ringelberg *et al.*^14^

Ringelberg *et al.* used data for 44 Florida communities to estimate CWF costs. (Florida’s phosphate industry is the largest U.S. producer of fluoridation chemicals.)^15^,^16^ Ringelberg *et al.*’s improved estimates included costs for bulk storage and containment, labor, and opportunity costs of capital investment, and were based on a larger number of communities than previous estimates.^13^ The estimated average cost increased from $0.49 PPPY^13^ to $1.25 PPPY.^14^ With phrases such as “allowable initial one-time costs … were documented by copies of actual invoices for equipment and services” Ringelberg *et al.*^14^ appears detailed and based on actual data. However, these invoices were obtained from the Florida public health dental program, which has the authority to approve costs for communities seeking state grants to implement CWF,^17^ and thus reflect costs allowed by the state dental program rather than actual costs.

Ringelberg *et al.*^14^ used a 15-year life, with no remaining value, for initial implementation costs, and used 2.4% of the initial costs to calculate the maintenance and repair costs. Labor costs provided by CDC’s fluoridation engineer were based on 1 hour per day for all systems and rates of $7 per hour for small systems and $9 per hour for medium and large systems. (Note that, in contrast, Input (d) in Table 2 uses $18 per hour to calculate CWF benefit.) We will show that this is a simplistic and unrealistic view of what is involved in CWF operations.

### Reality on the ground

In 2010, amid a budget crisis, the City of Sacramento, CA, instructed all departments to review programs and services. Mr. Marty Hanneman, then Director of the Department of Utilities, wrote in a memo to the City Council:^18^

The City of Sacramento has been fluoridating its water supplies just over 10 years. Within that time, the actual cost of operating and maintaining the fluoridation systems has proven to be considerably more than the initial estimate. … The fluoridation infrastructure at the E.A. Fairbairn Water Treatment Plant is overdue for replacement and will be very expensive to replace. … Fluoridating water is a very costly and labor intensive process and requires constant monitoring of fluoride concentrations to ensure proper dosages. … The chemical is very corrosive, so all equipment that is used in the fluoridation process has a very short life expectancy and needs to be replaced frequently. … but also causes frequent and complex system failures.

This was echoed by Mr. René Fonseca of Carroll-Boone Water District in Eureka Springs, AR, which was required by a 2011 State mandate to begin CWF (Fonseca, 2012, private communication):

All of our chemical feed systems require regular maintenance which is routine but fluoride feed equipment often requires replacement and more frequent attention. … I have toured plants and seen in trade publications deteriorating pipes, steel doors and casing, electrical components, etc. There are millions of dollars spent yearly on infrastructure damage caused by fluoride in our industry.

The realities expressed in these two quotes are not the exceptions. A water plant manager in Alberta, Canada, complained that the fumes from the acid etched the glass, paint, and computer screens of the water treatment plant.^19^ Seven years after CWF began in 2001, Riverton, Utah, spent nearly $1.2 million for two new buildings “to get fluoride out of electrical and pump area.”^20^

Several incidents of fluoride overfeeds at water-treatment plants have been investigated. Gessner *et al.*^21^ described an accident that occurred in Hooper Bay, Alaska, in 1992, in which 296 residents suffered acute poisoning and a 41-year-old man died. Petersen *et al.*^22^ reported on an overfeed incident in a residential Connecticut community in 1986. The fluoride caused gastroenteritis in 33% of those who drank the water and itching and skin rashes in those with dermal contact; the acidity leached copper from domestic plumbing. Penman *et al.*^23^ investigated an outbreak of acute poisoning caused by a fluoride overfeed in a small rural community in Mississippi in 1993. Several people became ill and connected the onset of their illness to drinking tap water at the same restaurant. A community survey was performed, and the authors concluded that approximately one third of households in the town may have been affected, though the extent remains unknown.

Akiniwa^24^ examined seven events of acute fluoride poisoning related to the fluoridation of drinking water that have been reported in the U.S. He estimated from these reports that acute fluoride poisonings have occurred at doses of 0.1–0.8 mg fluoride per kg of body weight. One fatal fluoride intoxication caused serious illness in 12 patients, 3 of whom died, in a hospital hemodialysis unit in Chicago in 1993.^25^ Caused by failure of a widely used deionization system, this event would not have been catastrophic had the water not been fluoridated.

Other incidents reported in local media have included injuries to water plant workers, massive evacuation around an interstate highway, damages to water pipes or concrete floors, and environmental hazards to fish and ground. A number of these incidents are cited in Appendix 3.

An economic evaluation taking a societal perspective should have considered the societal costs from these inevitable consequences of CWF. However, comprehensive data needed to estimate such costs are lacking, because the government agencies that should track these incidents appear to have a conflict of interest in protecting and defending the CWF policy (e.g. Florida;^26^ Layton;^27^^iv^ Appendix 1). Nevertheless, evidence presented here demonstrates that Ringelberg *et al.*^14^ were unrealistic even considering only the direct costs of CWF to a water system.

### Real-world estimates

In late 2010, Black and Veatch Corporation (Overland Park, KS USA) was retained through a competitive bid process to perform an objective evaluation of the fluoride program of the city of Sacramento, CA. After a comprehensive and detailed review, the study^28^ observed that immediate and future upgrades would be needed to continue fluoridating and to achieve modest operational efficiency improvements. Noting that Sacramento’s operational costs were within industry practice, the report developed detailed cost estimates and gave a different picture from Ringelberg *et al.*,^14^ e.g. the labor cost was set at a rate of $100 per hour, in contrast to the $7/$9 per hour labor rate from the CDC. The city’s engineer, Mr. Brett Ewart, explained (Ewart, 2012, private communication):

The 100/hr. is a hybrid rate used to represent the large variety of machinists, electrical staff, water quality staff, management, etc., that work on the program. The amount of staff time (and type of staff) dedicated to the fluoride program is flexible. Some maintenance activities are generally fixed, others are reactionary and difficult to predict in advance. The rate would include the employees’ salary, benefits, and overhead to perform the work.

Sacramento’s water system consisted of the following: One large treatment plant supplying 44% of the water, whose fluoridation system had already been updated in 2007; a second large treatment plant (Fairbairn WTP) supplying 42% of the water, whose fluoridation system was in need of replacement; and 27 wells supplying 14% of the water, and whose fluoridation systems also required updates.^29^ The overall cost estimates provided by Black and Veatch for the needed replacement and updates, annualized using a 2.5% discount rate over a 20-year planning horizon,^v^ were $1 million for the 27 wells and $464,000 for the Fairbairn WTP.^29^ The cost projection for the Fairbairn WTP is applicable for large water treatment plants, while the cost projections for the well upgrades are applicable for small systems.

To calculate the PPPY costs, we allocated the total population of Sacramento, 466,000 people (2010 U.S. Census), to the 27 wells and to the Fairbairn WTP using the percentages of total water supplied of 14% and 42%, respectively. The allocated populations are 65,000 and 196,000, respectively. Dividing the total costs by population and number of injection sites, we obtain a cost estimate of $15.38 and $2.37 PPPY for a single-injection point water system serving 2,400 and 196,000 people, respectively. (Systems serving 2,400 people are not rare. Of the 44 systems in Ringelberg *et al.*^14^ three systems had smaller populations and seven systems had smaller populations per injection site.)

We considered whether to adjust for the cost of living in Sacramento and determined that there was no need. The cost of living for Sacramento is 8% higher than the U.S. average.^31^ This differential, however, is easily offset by other considerations, e.g. the use of a 2.5% instead of 4% discount rate. The cost projection also assumes that the Health Department continues to waive a requirement for certain standard equipment. In addition, actual bids for construction may turn out to be much higher than the engineer’s estimates.^32^,^33^ Finally, it was unknown whether implementing the recommendations would solve the city’s fluoridation issues.^29^

A small water system serving more than 2,400 people is expected to cost less than $15.38 PPPY. Similarly, many large systems serve less than 196,000 people and are expected to cost more than $2.37 PPPY. (Note that large water districts serving more than 196,000 people will not necessarily cost much less than $2.37 PPPY, because such water districts often have multiple treatment plants and/or auxiliary wells, which make them equivalent to a smaller single-injection point system). Therefore, reasonable cost estimates for the smallest (<5,000 people) and largest (>20,000) systems in Table 1 would be about $10 and $3 PPPY, respectively.

Strictly speaking the annual cost projections provided by Black and Veatch are 20-year financing costs. At the end of the 20-year period, components such as new buildings may still have value. However, given the ability of the chemicals to degrade concrete (Appendix 3 items 17 and 19), significant annual maintenance and repair costs after the financing period are expected. In addition, circumstances could require a water system to implement major infrastructure changes to their fluoridation facilities. Sacramento is such an example. Despite implementing fluoridation comparatively late (around 2000), the city has already endured major infrastructure adjustments and is considering more, long before the 20-years projection period. Finally, it is possible that a system may discontinue CWF; in that case, buildings constructed specifically for CWF may hold little value.

### Other estimates

The Black and Veatch report cited above is valuable in that it is recent, comprehensive, detailed, and authored by a firm that has consulted on other fluoridation programs. In general, reliable cost information for CWF programs is difficult to obtain, and information provided in response to a request is often limited to the cost of the fluoride chemicals. In Table 4, we present additional cost information and estimates collected from various sources.^20^,^32^^–^40 The majority of these are cost estimates prior to implementation; New York and some Utah figures show actual costs. Costs are reported either for implementation (*I*) or for annual operation and maintenance (*O*). For convenience, we calculate a PPPY cost by annualizing the implementation cost (*I*) using a 4% discount rate over 15 years^vi^ (meaning $100 annualizes to $8.65) and normalizing the total, i.e., dividing the annualized *I* plus *O*, if available, by population. (Population figures are taken from the CDC website or the U.S. Census Bureau if they are not reported in the source article.)

**Table 4:** Examples of fluoridation cost estimates

Water districts	Year Est	Pop (in thousands)	Reported implementation (I) and annual operation and maintenance (O) costs	PPPY ($) 15 years, 4% (I)
Napa, CA^34^	2003	77	I: $1M; O: $150,000	3.07
New York, NY^35^	2008	8,350	I: $12.57M (2 plants); O: $11.14M (chemicals)	1.45
San Jose, CA^36^	2011	1,000	I: $23M; O: $1.732M (Wells only)	3.72
Watsonville, CA^32^	2011	51	I: $50/person; O: $4/person	8.33
Portland, OR^37^	2012	900	I:$3.5M–$7.6M, O: $575,000	0.98–1.37
Carroll-Boone, AR^33^,^38^	2012	25	I: $894,000–$1.23M	3.09–4.26^†^
Davis, CA^39^	2013	67	I: $1.1M–$2.4M; O: $228,800–$240,700	4.84–6.69
Riverton, UT^20^	2000	35	I: $90,000 (estimate)	0.22^†^
	2001		I: $200,000 (actual)	0.49^†^
	2008		I: $1,174,278 (actual w/2 new buildings)	2.90^†^
Jordan Valley, UT^40^	2000	82	I: $56,000–$2.1M (estimates)	0.06–2.22^†^
	2004		I: $2.45M; O: $297,000 (actual)	6.21

^†^Estimates do not include operation and maintenance costs.

Many of the cost estimates shown in Table 4 are incomplete or partial, or the values are underestimated. Several (denoted with †) do not include operation and maintenance (O&M) costs. The New York numbers consist of costs to rehabilitate CWF facilities in two plants, and only chemicals are included in O&M. The San Jose numbers provided in a Black and Veatch study were for wells that provide only half of the water for the city, which imports the other half. The preliminary estimates for Napa, CA are from about the time that Sacramento began its fluoridation program and probably suffer from similar underestimates of costs.^18^ The estimates for Portland, OR were provided by the Water Bureau after a meeting with representatives from the CDC and the organizations pushing to fluoridate the city. The $575,000 O&M figure appears unrealistic — Sacramento already paid over $400,000 back in fiscal year 2008/2009 for hydrofluorosilicic acid (HFSA) to fluoridate 86% of their water; this translates to about $1 PPPY for the cost of HFSA alone. In addition, the O&M estimate excluded costs of additional caustic or other corrosion control chemicals to bring the pH back to an appropriate level, and the cost of additional capital improvements needed to mitigate water quality impacts were not included in the estimated capital costs.^37^

Community water fluoridation proponents have a poor track record for cost estimates. For example, the county health board of Davis County, UT, provided a cost estimate of $1.38–$2 PPPY prior to a vote in 2000, but the true implementation cost was $4.29 PPPY.^41^ This is also seen in the estimates/observed figures for the two Utah systems in Table 4. In 2001, Arkansas state legislators passed a state mandate to fluoridate community drinking water. They were partially motivated by an offer from Delta Dental of Arkansas to donate $500,000 total toward startup costs for the 32 water systems affected.^42^ Later Delta Dental pledged $2 million for 34 systems and soon found itself needing to raise another $6–$10 million.^43^ (State mandates in California and Arkansas both require the initial implementation costs be funded by outside sources.)

Overall, reported costs of CWF are consistent with our real-world estimates and not with those estimates^10^,^14^ commonly cited by fluoridation proponents.

### Costs of dental fluorosis

Griffin *et al.*’s Assumption (4) in Table 2, that the adverse effects of CWF are negligible,^10^ is common to most cost-benefit analyses of CWF. It is inexplicable that neither Griffin *et al.*^10^ nor other similar studies (Appendix 2) mention dental fluorosis, defective enamel in permanent teeth due to childhood overexposure to fluoride.^44^,^45^ Community water fluoridation, in the absence of other fluoride sources, was expected to result in a prevalence of mild-to-very-mild (cosmetic) dental fluorosis in about 10% of the population and almost no cases of moderate or severe dental fluorosis.^46^ However, in the 1999–2004 NHANES survey, 41% of U.S. children ages 12–15 years were found to have dental fluorosis, including 3.6% with moderate or severe fluorosis.^47^

As an increased prevalence of dental fluorosis became evident, there were attempts to shift attention to other sources of swallowed fluoride, such as toothpaste.^48^ However, 1/4 liter (or about 8 oz) of fluoridated water at the “optimal” concentration of 1 mg/l contains the same amount of fluoride as a bead of toothpaste (0.15% w/v fluoride ion) 0.68 cm in diameter. Regarding other sources of ingested fluoride, Szpunar and Burt^49^ state that the factor that differentiates the studied communities with respect to the prevalence of caries and fluorosis is the fluoride concentration in the community water supply.

Dental fluorosis had been dismissed as cosmetic by CWF promoters and government agencies in the U.S. until the National Research Council (NRC) concluded that “severe dental fluorosis” qualified as an adverse health effect due to increased risk of caries and loss of dental function.^44^ When an economic evaluation is framed as having a societal perspective, it should include effects that result in social costs, regardless of whether the effects are cosmetic or systemically harmful. In a later paper, Griffin *et al.* indicated that some people may want “esthetic restorative procedures” to treat fluorosis, but treatment costs were not estimated.^50^ We next provide a high level estimate of the minimal costs of treating dental fluorosis.

Dental fluorosis is classified by the severity of the discoloration, the presence of pitting, and the extent of the tooth surfaces affected.^44^,^45^vii Although bleaching and microabrasion can be used to improve the appearance of milder cases of fluorosis, moderate and severe dental fluorosis can require extensive treatment to improve the cosmetic appearance and prevent further loss of enamel.^44^,^45^ Treatment options include applications of veneers or crowns. Porcelain veneers may cost more than composite resin veneers ($800–$2,500 vs. $250–$1,500), but they require less frequent replacement (10–15 vs. 5–7 years).^52^,^53^ Crowns are “usually used as a last resort because they can be a threat to tooth vitality.”^44^

For this analysis, we assume that each moderate or severe fluorosis tooth receives a porcelain veneer treatment. We further assume that a child with the condition gets the first treatment at age 13.5 years, and the veneers are replaced every 12 years. The lifetime cost of a veneer is calculated using equation (1), except the $72 is replaced by the cost of a veneer, for which we use a lower-end number of $1,000. This gives a lifetime cost of $2,217. Dean’s Enamel Fluorosis Index, the most widely used classification of dental fluorosis, is assigned on the basis of the two most-affected teeth.^44^ Thus, the lifetime cost of veneers for a child with moderate or severe fluorosis would be at least $4,434.

Beltrán-Aguilar *et al.*^47^ reported that 3.6% of U.S. children ages 12–15 years in 1999–2004 had moderate or severe dental fluorosis, but did not provide information on the fluoridation status of the affected children. At most about 60% of the U.S. population received fluoridated water during the time period when these children were susceptible to development of fluorosis.viii Both the prevalence and the severity of dental fluorosis are correlated with the fluoride concentration in drinking water.^45^,^49^,^55^ If all of the cases of moderate and severe dental fluorosis occurred in fluoridated rather than nonfluoridated areas, then at least 6% of children in fluoridated areas would have moderate or severe fluorosis.^ix^ For our calculations, we have assumed that 5% of children in fluoridated areas have moderate or severe fluorosis. From Table 3, the percentage of children at age 13.5 years is about 20.4%/14 = 1.46%. Thus the minimum cost of treating dental fluorosis is estimated to be $4,434×1.46%×5% = $3.24 PPPY.

### Other costs

There are other costs missing from the conventional cost-benefit analyses of CWF (Appendix 2). The NRC’s 2006 report on fluoride exposures and toxicity found that the U.S. Environmental Protection Agency’s (EPA) drinking water standard for fluoride was not protective of human health.^44^ The NRC did not evaluate CWF for safety or efficacy, but the report showed that the average fluoride exposures associated with adverse health effects are within the expected range of fluoride intake for populations with fluoridated water, especially for infants, young children, and people with high water intake.^44^,^56^^x^ Peckham and Awofeso’s recent review specifically concluded that fluoridation has “significant costs” in relation to adverse effects on human health, although these costs were not quantified.^57^

Health risks to water plant operators are not included in most discussions of CWF, but these individuals may receive substantial occupational exposures to fluoride if the safety infrastructure or training is not adequate or if equipment malfunctions.^58^,^59^

Most of the fluoridation chemicals used in the U.S. are byproducts of the phosphate fertilizer industry in North America or Asia.^15^,^16^,^60^ Since only a small percentage of municipal water is actually consumed by people, the practice results in wide dispersion of a regulated pollutant into the environment via local water districts. Fluoride pollution may result in serious ecological risks to aquatic organisms.^61^

Fluoride is regulated by the U.S. EPA as a contaminant in drinking water^62^ and as an air pollutant.^63^ A number of fluoride compounds are considered hazardous substances with assigned Reportable Quantities.^64^ In addition, fluoridation chemicals often contain other regulated contaminants.^5^^–^8 Hirzy *et al.*^65^ estimated that the typical concentration of arsenic in the major fluoridation chemical (HFSA) could be responsible for several excess lung and bladder cancers per year in the U.S. and the consequent costs of treatment.

Political costs have at times been acknowledged but not included in CWF analysis.^10^ This category goes beyond costs associated with fluoridation referenda to include government expenditures for promoting fluoridation programs, costs associated with lobbying elected officials on this issue, legal challenges to fluoridation programs, and possible personal injury litigation involving workers or members of the public.^66^^–^70

There are also costs associated with avoiding fluoridated tap water, either by need or by choice. These are all societal costs of CWF that should not simply be excluded or assumed negligible without examination.

## BENEFITS

The primary benefit attributed to CWF is prevention of caries, although a major review in the United Kingdom reported no relevant studies of “evidence level A (high quality, bias unlikely)” and expressed surprise that little high quality research had been undertaken.^71^ Caries prevention is commonly assessed in terms of a reduction of decayed, missing, or filled teeth (DMFT), DMF tooth surfaces (DMFS), or their variations.^xi^ Estimation of averted caries is obviously central to a cost-benefit analysis.

Griffin *et al.*^10^ relied on the theory that caries averted by CWF can be considered in terms of two factors as shown in the following equation
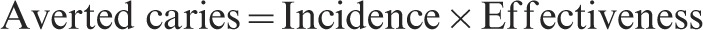
(2)where Incidence is the per person annual caries increment without CWF, and Effectiveness is the percentage reduction in caries due to CWF.

Before we explain and critique how Griffin *et al.*^10^ derived their values for Incidence and Effectiveness, it is worthwhile to examine the concepts of incidence and effectiveness in the context of CWF.

### Incidence

Griffin *et al.*^10^ treat the reported caries incidence in selected nonfluoridated areas as the caries incidence in the absence of CWF. However, they have not accounted for the decline in caries rates over time apart from CWF or the variability in caries rates among various areas, independent of CWF.

It has been known for decades that tooth decay prevalence has been declining in developed countries regardless of CWF status, i.e., the “secular decline”.^11^ Diesendorf^72^ listed over 20 studies which reported substantial temporal reductions in caries in unfluoridated areas. In many cases, the magnitudes of the reduction were comparable to those attributed to fluoridation in some fluoridated areas; it was also pointed out that fluoride toothpaste or supplement could not have accounted for many of the reductions.

That fluoride is not needed for dental health is not surprising. A 1952 NRC report^73^ described studies reporting that the teeth of ancient peoples and modern primitive peoples were relatively free from dental caries, in a striking contrast to the teeth of modern people. However, primitive peoples had increased rates of caries when brought into contact with a modern diet. This is consistent with the fact that caries are rare in animals in the wild. Finn^73^ also described the significant geographic and temporal variability of caries prevalence, citing Hagan^74^ for demonstrating how caries prevalence may vary within narrow geographic limits, as well as fluctuating within the same area from time to time.

Hagan^74^ studied 12 communities in Georgia, including 24,092 children, and reported the following by community: The average annual caries increments were 0.18–0.90 for children up to 16 years old; the DMFT ranged from 0.40–2.44 at age 7 years to 1.41–10.64 at age 16 years; the percentage of children with at least 1 DMFT ranged from 23–77% at age 7 years to 58–100% at age 16 years. The ranges of DMFT for a given age in these pre-CWF situations approach or exceed the differences reported between fluoridated and nonfluoridated locations in more recent years. For example, Heller *et al.*^55^ reported mean DMFS values ranging from 2.53 (0.7–1.2 ppm F) to 3.08 (<0.3 ppm F), with a mean DMFS of 2.75 for the entire sample (18,755 U.S. schoolchildren ages 5–17 years with a history of a single residence). The percentage of caries-free children ranged from 52.5% (>1.2 ppm F) to 57.1% (0.3–0.7 ppm F), averaging 54.6% for the entire sample. McDonagh *et al.*^71^ reported that, among 15 studies analyzed, the mean differences in dmft or DMFT ranged from 0.5 to 4.4 (median 2.25).

Other historical data contradicting the idea that fluoride is needed for dental health have been reported. Using data from New Zealand Health Department records of 5-year-olds’ tooth decay from 1930 to 1990, fluoridation coverage, and fluoride toothpaste sales, Colquhoun^75^ showed that the dramatic decline in tooth decay started long before water fluoridation, fluoride toothpaste, or application of fluoride. Another paper noted that the DMF rate in children ages 12–15 years in Taiwan was as low as 1/3 to 1/6 of that in children of the Western countries where water fluoridation had been in effect for 8–11 years.^76^

Studies that attributed differences in tooth decay rates between selected communities to CWF may have only observed these geographic or temporal variabilities, independent of any effect of CWF. Other studies (see Appendix 5) found that nonfluoridated cities also experienced rapid reductions in tooth decay rates without installing CWF, even though these cities had previously been compared with fluoridated cities as evidence that CWF reduces caries. Hence the concept of a no-fluoridation caries incidence rate has little meaning.

### Effectiveness

Griffin *et al.*^10^ derived their estimate of effectiveness from Brunelle and Carlos,^77^ who reported on the second of two large-scale National Institute of Dental Research (NIDR) surveys, completed in 1980 and in 1987, respectively. Each survey sampled and examined approximately 40,000 U.S. school children aged 5–17 years.

Community water fluoridation effectiveness has been variously reported in the literature. The unit of measure can be variations of DMFT, DMFS for permanent teeth, the corresponding measures for deciduous teeth, or the percentage of children with no caries. They could be for a single age or for an age range. Information about length of exposure to CWF may or may not be included. Study parameters are often poorly defined and confounding factors not typically examined.

Often a percentage value is produced from some relative differentials and referred to as CWF effectiveness, despite that the percentages come from different situations. Some may argue that since all the different kinds of studies point to similar ranges of effectiveness, it is proof that the effectiveness estimates are robust. However, the premise of this argument is false.

*First: Units.* Units of measures do affect the results. An independent investigation of the 1987 NIDR data using DMFT instead of DMFS led to the conclusion of no effectiveness.^78^ When asked about results for teeth, Brunelle was quoted to have said that they “are in a box somewhere” and she “could not remember what exactly the results were” and that the decay rate for teeth “is rather low so that there is very little difference in most anything.”^79^ Truman *et al.*^80^ estimated effectiveness in units of teeth from data reported in a number of studies (Table 5) even if a study reported data in both teeth and surfaces.

**Table 5:** The studies, the age of children examined, group placed, and number of estimates calculated by Truman *et al.*^80^ to evaluate CWF effectiveness

Study	Age	Group/no. Est.
Arnold and Dean^93^	4–15	A-On/4
Beal and James^94^	5	A-On/2
Beal and Clayton^95^	5,8,12	A-On/4
Loh^96^	7–9	A-On/2
Evans *et al.*^97^	5	A-On/3, B-On/1
Guo *et al.*^76^	4–15	A-On/2, B-On/3
Künzel and Fischer^98^	6–15	A-On/4, A-Off/2
Attwood and Blinkhorn^99^	10	A-Off/1
Kalsbeek *et al.*^100^	15	A-Off/2
Brown and Poplove^101^	14–17	B-On/4
Fanning *et al.*^102^	3–6	B-On/3
Hawew *et al.*^103^	6,12	B-On/4
Provart and Carmichael^104^	5	B-On/2
Rugg-Gunn and Nicholas^105^	5	B-On/3

Studies reporting results in teeth were more common in the past. The focus shifted toward surfaces as the prevalence of caries dropped and caries became concentrated in a small subset of the population.^81^ Measuring caries in units of surfaces gives heavy weight to the small percentage of people with high levels of decay.xii

*Second: Lengths of exposure.* There are two relevant exposures: exposure to carious influence and exposure to CWF.

Exposure to caries is determined in part by the time a tooth erupts. Usually age is used as a surrogate for the length of this exposure. If a study examines subjects of a range of ages and one effectiveness number is to be presented, which age is selected or how different age groups are weighted to calculate an average can produce different results. Appendix 4 provides examples of studies showing differences in caries experience that were attributed to CWF exposures, when the results may be better explained by differences in age distributions of the populations being compared.

Exposure to CWF is often handled by comparing only those with lifetime exposure to those with no exposure. However, if a result is contingent on excluding partial exposure it weakens the argument for CWF as a public policy. More importantly, this approach introduces a probable bias if the two exposures (to caries and to CWF) are not independent. Evidence indicates that ingested fluoride may delay tooth eruption,^44^,^45^,^85^ which would affect caries scoring by giving the appearance of less decay for a given age.^44^,^45^ Komárek *et al.*^86^ used data for actual tooth eruption time and found no convincing effect of fluoride intake on caries development. Weaver^87^ indicated that “the caries inhibitory property of fluorine seems to be of rather short duration,” consistent with a delay in the exposure of permanent teeth to a cariogenic environment.

*Third: Methods.* The methods of determining an effectiveness value are even more problematic, especially in regard to policy references. This is best demonstrated by an examination of Truman *et al.*,^80^ which was co-authored by Griffin, other CDC personnel, and a Task Force appointed by the Director of the CDC. The Task Force was established by the U.S. Department of Health and Human Services (HHS) in 1996 to provide recommendations for community preventive services, programs, and policies. Reported in 2000, the findings of the Task Force’s systematic review^88^ became the main results of Truman *et al.*^80^ on CWF effectiveness, as well as the basis for Healthy Peoplexiii2010’s goal of increasing CWF in the U.S. to cover 75% of the population.^91^ Healthy People 2020^92^ continues with a goal of increasing coverage to 79.6%.

Truman *et al.*^80^ based their conclusion on 14 studies in three groups (Table 5):^76^,^93^^–^105

- Studies starting or continuing CWF with before and after measurements (Group A-On)
- Studies stopping CWF with before and after measurements (Group A-Off)
- Studies starting or continuing CWF with only post measurements (Group B-On)

They calculated a number of “estimates of effectiveness” from the studies using two formulas, one for Group A (before-and-after) and one for Group B (post measurements only). The measures were mostly DMFT or dft.

The median of estimates was taken to represent the CWF effectiveness for each study type; the results were 29.1% for Group A-On, 50.7% for Group B-On, and 17.9% for Group A-Off. (The 29.1% and 50.7% figures were presented by the Task Force.)^88^ With these numbers the authors concluded “strong evidence shows that CWF is effective.” This conclusion is not valid. We describe three areas of problems below (details provided in Appendix 5).

*Selection of studies*: Studies of higher quality and relevance such as the NIDR surveys or other U.S. studies were not included. Many studies on the effect of cessation of CWF (Group A-Off) were omitted even though this group had only three studies. Not all included studies are relevant for CWF or meet the stated criteria.

*Selection of estimates*: The number of estimates selected from each study appears arbitrary. Fewer estimates were selected from large-scale studies reporting findings in detail than from small studies reporting few findings. Sometimes the selected estimate did not fit the group it was placed in. Selection of arbitrary numbers of estimates from an arbitrary set of studies does not lead to confidence in the reported median.

*Selection of formula*: Within the limited set of studies and estimates selected, the authors failed to apply their formula consistently. In addition, the results from the application of the formula can be misleading. Upon examination of the data, some purported positive outcomes are revealed as purely an artifact of the formula — the never-fluoridated communities had a dramatic reduction in caries without the help of CWF.

### The incidence and effectiveness in Griffin *et al.*^10^

Three estimates for Incidence were compiled from several unrelated sources while three estimates for Effectiveness were derived from a single source. They are paired by magnitude and substituted in Equation (2) to arrive at three cases of averted DMFS as shown in Equation (3).
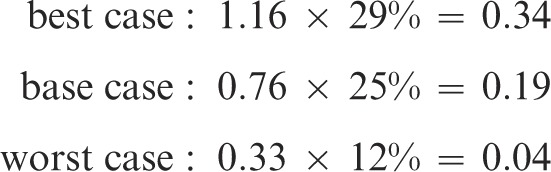
(3)

The base-case averted DMFS of 0.19 is the key input (g) in Table 2. (Note that not all studies cited by the authors measured DMFS, and the differences were not always pointed out.) We next examine how the numbers on the left-hand side were derived.

#### The Incidence

Griffin *et al.*^10^ obtained three sets of annual caries increments in nonfluoridated communities as Incidence; they are reproduced in Table 6. The sources were, respectively, published studies cited in Garcia,^13^ the National Survey of Oral Health (NSOH) in U.S. Schoolchildren and a separate NSOH in Employed Adults and Seniors, and the First and Third National Health and Nutrition Examination Survey (NHANES I, 1971–1974 and NHANES III, 1989–1994).

**Table 6:** Griffin *et al.*’s estimates of annual caries increment (tooth surfaces) from selected studies, by age^10^

	Age (year)			
Source	6–17	18–44	45–65	Avg.
Published studies (best case)	1.40	0.83	1.24	1.16
NSOH (base case)	0.77	1.09	0.43	0.76
NHANES (worst case)	0.49	0.49	0.00	0.33

For the best case, the authors used the controls in Garcia’s review^13^ of published studies of clinical and community trials. For the base case, the incidence for children was imputed by dividing the difference in mean DMFS for 6-year-olds and 17-year-olds living in communities without fluoridation by 11. Unrelated to the children’s survey, the adult NSOH survey was measured in DFS (without M, missing surface) and was not stratified by community fluoridation status. Hence, they imputed the incidences by using the least fluoridated region (Pacific). They scaled the mean DFS by the ratio of average numbers of teeth in the two age points to adjust for missing teeth. They also added root caries incidences from other studies. For the worst case, the authors imputed the incidence using data from two NHANES surveys, which did not report fluoridation status. A major difference from the base case was that they tried to use data on the same birth cohort over time. Additional adjustment was applied because earlier NHANES data measured DFT instead of DFS.

The source of the best case, Garcia,^13^ was the basis for discussion of CWF in the 1989 Michigan Workshop. Workshop participants were critical of the numbers. “Most work groups felt that the estimates of caries incidence in Garcia’s report were generally too high and reduced them by several decimal points, though some reduced them further.”^12^ Griffin *et al.*^10^ also stated in their discussion that the samples were probably not representative of the general population. Thus, the best case is invalid.

Griffin *et al.*^10^ also admitted that the base case was overestimated. They remarked that, given the secular decline in caries, using cross-sectional data to impute caries increment from the NSOH would overestimate current increment. Secular decline^11^,^72^ refers to the widespread decline in caries observed in nonfluoridated areas. It means that when a 6-year old living in a nonfluoridated area today grows up to be 17 years old, he will likely have fewer caries than his 17-year old neighbor has today. Thus using the latter to represent the former (cross-sectional data) overstates the incidence of caries.

#### The Effectiveness

As with the Incidence, Griffin *et al.*^10^ presented three cases for Effectiveness, all essentially from Brunelle and Carlos.^77^ The 1987 NIDR Survey examined 39,206 children, of whom about 92% had complete residence histories. Brunelle and Carlos^77^ analyzed data from 16,398 children with either lifelong exposure or no exposure to CWF and presented mean DMFS by age (see Table 12) and by region (Table 7). The national averages from this subset of data showed a difference of 0.6 DMFS, or 18%, between the two exposure groups. By further restricting their sample to a subset of 5,954 children (reportedly by removing all data points with any supplemental fluoride exposure), the 18% difference was raised to 25%. No age or regional distribution was shown for this restricted set of data. Griffin *et al.*^10^ took this 25% as the base-case Effectiveness.

**Table 7:** Mean DMFS of each U.S. region by CWF status (1986–1987) from Brunelle and Carlos^77^

Region	Lifelong exposure	No CWF exposure	Population with CWF (%)	Relative Diff (%)
I	3.11	3.45	55	9.9
II	3.08	3.42	49	9.9
III	2.86	2.69	74	–6.3
IV	2.75	3.60	54	23.6
V	2.49	2.71	59	8.1
VI	2.36	3.07	34	23.1
VII	1.42	3.61	19	60.7
U.S.	2.79	3.39	53	17.7

DMFS: decayed, missing, or filled tooth surfaces; CWF: community water fluoridation.

Brunelle and Carlos^77^ ignored 58% of the total data (or 55% of those with complete residence histories), despite that partial exposure data from this national survey can be analyzed and are informative.^78^ It is therefore questionable if the 18% reduction in DMFS represents the findings of the survey. Even more troublesome is the 25% adopted as the base-case Effectiveness, as it ignores 85% of the survey data.

The best- and worst-case Effectiveness, 29% and 12%, respectively, were supposed to be calculated from the best three and the worst four effective regions. However, the worst four regions (I, II, III, and V in Table 7) would average closer to 6% than 12% using regional population data found elsewhere.^106^ It appears that Griffin *et al.*^10^ may have removed Region III (Midwest) from the calculation given the comment: “The negative effectiveness value in the Midwest may have been due to small sample size because few children living in this region actually received nonfluoridated water.” This criticism would equally apply to the highest-effectiveness Region VII (Pacific), as few children in this region received fluoridated water, but it was not considered a problem.

### Lack of evidence for adults

Assumption (2) and Input (h) in Table 2 assume the same CWF benefit to age 64 years, despite that estimates of Effectiveness were derived from a children’s survey. Two adult studies^51^,^107^ were cited to support this extrapolation. However, the data presented in Grembowski *et al.*^107^ do not support its conclusion, and Eklund *et al.*^51^ appear to be mis-cited in addition to the fact that the concentrations involved, 3.5 versus 0.7 mg/l, are irrelevant to an evaluation of CWF. We examine each of these studies below.

That few adult studies are available has been noted elsewhere. Garcia^13^ stated that very limited information exists in the literature about caries incidence in adults, and Newbrun^108^ identified only seven adult studies; he commented that very few acceptable data exist and that the comparison was either between those living in low-fluoridated and high-fluoridated (greater than optimal) communities or between those living in optimally fluoridated and high-fluoridated communities. Thus, it is not surprising that Truman *et al.*^80^ included “What is the effectiveness of CWF among adults aged ≧ 18 years?” among important unanswered questions.

More recently Slade *et al.*^109^ presented an analysis of Australian data from a 2004–2006 survey, and Griffin *et al.*^110^ did a meta-analysis of several earlier studies. We examine these papers in detail in Appendix 4. Among other problems, both articles (and several studies included in the latter) failed to properly account for different age distributions.

#### Grembowski *et al*.^107^

This study examined Washington state employees and spouse-dependents aged 20–34 years living in Olympia, Seattle, or the Pullman, WA/Moscow, Idaho area. The data presented in this study are reproduced in Table 8.

**Table 8:** Average number of decayed and filled surfaces by period of fluoridated exposure in lifetime from Grembowski *et al.*^107^

Period of fluoridation exposure in lifetime	No. of adults	Average age	College degree (%)*	Average DFS
No exposure in lifetime	226	30.4	35	27.9
Pre-eruptive exposure patterns (ages 0–5 only or ages 0–14 only)	40	30.1	40	20.0
Post-eruption exposure patterns (ages 15–34 only)	266	30.7	78	22.2
Exposed most of life	63	31.0	72	15.7

*Includes those with a college degree and those with graduate work or a graduate degree.

Griffin *et al.*^10^ paraphrased Grembowski *et al.*^107^ claiming that the average 30-year-old adult with continuous lifetime exposure to fluoridated water had 8.7 fewer decayed or filled surfaces, or a 31% reduction compared with 30-year-old adults with no CWF exposure. However, based on the data (Table 8), it is unclear how these figures were estimated.

There are additional problems with Grembowski *et al.*^107^ For example, it was stated that “1,066 … formed the data base for this analysis”; but the paper shows results for only 595 participants, and makes no mention of the other 471 participants. In other words, 44% of the data are unaccounted for.

Grembowski *et al.* described calculating the years of fluoridation exposure for the age ranges: 0–5, 6–14, 15–19, and 20–34 years, to “explore systemic and topical effects.” However, Table 8 has a group described as having an exposure pattern, meaning exposure to CWF for the majority of time during the period of “ages 0–5 only or ages 0–14 only” — it appears to be a hastily created grouping to avoid showing results from the original design. Indeed only 40 adults were in this group, so that they had to qualify their conclusion that “exposure to fluoridated water during childhood has lifetime benefits” with “These results are tentative, however, because the pre-eruptive sample size was small.”

The four groups differed in their education levels as well as their fluoride exposure (Table 8), with the no-exposure group having the lowest percentage with a college degree. The CDC has reported that oral health disparities are associated with lower education level.^111^xiv Although Grembowski *et al.* pointed out the difference in education level, they did not evaluate the possible impact of this difference on their findings.^107^

Grembowski *et al.* revealed that people in the nonfluoridated sites had less untreated decay than in the fluoridated sites. They also pointed out that the filled component of DFS is influenced by dentists’ treatment decisions. They noted that dentists in nonfluoridated areas may restore teeth in adults more frequently, and that use of identical treatment criteria would “slightly reduce” their estimates of fluoridation’s benefits.

They claimed to offer evidence that exposure to fluoridated water during childhood has a lifetime benefit and concluded that their findings provide support for health officials to continue and expand this public health program. Their data do not support the conclusion.

#### Eklund *et al.*^51^

This study examined the communities of Lordsburg and Deming, New Mexico, with fluoride concentrations of 3.5 and 0.7 mg/l in the drinking water supply, respectively. Subjects were approximately 30–60 years of age, had been born and lived at least the first 6 years of life in the city, and had an unequivocal water history. The main results were summarized in two tables, one for dental fluorosis and one for caries, reproduced in Tables 9 and 10, respectively.

**Table 9:** Number and percent of subjects by city and fluorosis classification from Eklund *et al.*^51^

Fluorosis	Lordsburg (*n* = 164)		Deming (*n* = 151)	
Normal			104	(68.9%)
Questionable			23	(15.2%)
Very mild	1	(0.6%)	17	(11.3%)
Mild	1	(0.6%)	2	(1.3%)
Moderate	37	(22.6%)	5	(3.3%)
Severe	63	(38.4%)		
Very severe	62	(37.8%)		

**Table 10:** Comparison of mean decayed, missing, or filled teeth (DMFT) and selected components by city and age of lifelong resident adults from Eklund *et al.*^51^

	Decayed		Missing		Filled		DMFT	
Age group	L	D	L	D	L	D	L	D
All	0.8	0.6	2.8	2.4	2.9	5.4	7.0	8.7
27–40	0.4	0.7	1.3	1.6	3.6	4.4	5.9	6.9
41–50	1.5	0.5	2.4	3.7	2.4	6.6	7.1	11.1
51–65	0.6	0.2	5.6	3.3	2.2	7.3	8.8	11.1

L: Lordsburg; D: Deming

Griffin *et al.*^10^ wrote that this work found adults who received a high fluoride concentration experienced 20% fewer carious *surfaces*. The 20% number was an interpretation from two numbers, 7.0 and 8.7, found at the upper right corner of Table 10. (Note: the unit of measure was teeth, not surfaces.) The authors, however, were less inclined to draw the kind of conclusion that Griffin *et al.*^10^ did. They wrote:

The picture is less obvious for dental caries. … The assessment of dental caries in an adult population is difficult. … First, it is often difficult to determine why missing teeth were removed. … Second, it is not possible to determine whether all filled teeth had a carious lesion as defined by the diagnostic criteria.

In contrast, they concluded that differences between the communities are “obvious and unequivocal” for dental fluorosis. Indeed, no one from Lordsburg escaped dental fluorosis and 76% of them were severe or very severe. At the lower concentration of 0.7 mg/l, Deming had 16% dental fluorosis, including some moderate cases.

Table 10 shows that the higher DMFT in Deming was due to a much higher filled component across all age groups. As with Grembowski *et al.*,^107^ Eklund *et al.*^51^ noted that the filled component is influenced by dentists’ treatment decisions. On the other hand, the oldest age group in Lordsburg had many more missing teeth, similar to other studies that found a relationship between high fluoride exposure and tooth loss.^112^,^113^

### Costs of dental treatments

Costs of dental treatments consist of dental fees and lost productivity. Griffin *et al.*^10^ used survey data for the dental charge,^114^ which may differ from the charge in a competitive market, and therefore not be representative of the resource costs. Assumption (6) holds that all fillings are single-surface fillings. This overestimates dental costs, since a three-surface cavity does not require three times more resources than a one-surface cavity requires, in terms of either time lost or dentist’s effort. In fact, the fees in the survey were $53.60 and $83.27 for one- and three-surface amalgam fillings, respectively.^114^ Griffin *et al.* used the U.S. average hourly wage for the productivity cost. Average hourly wage overestimates productivity cost, since another central argument for CWF is equity, i.e. it is supposed to be particularly beneficial to low-income people.

### Minimal corrections

In this section, we show how the defects in the derivation of CWF benefits, or gross savings, discussed above can be corrected.

#### Costs of dental treatments

The resource value of a treatment is best represented by the allowable charge from a widely accepted insurance fee schedule. Fee schedules may vary for a number of reasons, but the relative values among closely related procedures tend to be stable.

Table 11 shows the allowable charges for amalgam fillings from two large payers, one from a public payer^115^ and one from the largest commercial payer (private communication). The payments are not proportional to the number of surfaces involved, and Assumption (6) in Table 2 clearly overestimates the dental charges. Using these relativities and two assumptions a new gross savings estimate will be provided.

**Table 11:** Allowed charges and their relativities for amalgam fillings from two insurance fee schedules

Surfaces	Denti-Cal (CA Medicaid, $)	Delta Dental (San Diego area, $)
One	39 (1)	72 (1)
Two	48 (1.23)	87 (1.21)
Three	57 (1.46)	108 (1.50)
Four or more	60 (1.54)	118 (1.64)

Our first assumption is that the average number of decayed surfaces per filling is two and the average dental fee is about that of a two-surface filling. For example, a 40% : 30% : 20% : 10% distribution of one-, two-, three-, and four-or-more-surface fillings, respectively, produces such averages using the relativities in Table 11. Our second assumption is that each equivalent two-surface filling costs 1 hour in lost wages.

Brown and Lazar^114^ reported that there were more two-surface fillings than one-surface fillings in the 1990 survey and that the number of one-surface fillings has been dropping faster despite a vastly increased number of examinations. Since the more the distribution is weighted toward more-surface decays the less gross savings there are, our first assumption likely overestimates gross savings.

Using the more generous 1.23 factor from Denti-Cal to calculate a correction, the average cost per carious surface, $54+$18, in Step 1 is changed to
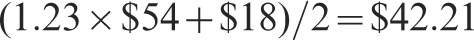
(4)

The $54 fee for a one-surface amalgam filling was based on a survey of about 5% of U.S. dentists in private practice.^114^ We argue that the allowed charge from a major commercial dental insurer better represents the true cost of resources, and we have an actual allowable charge of $72 from the San Diego area (Table 11). The cost of living in San Diego is 1.43 relative to the U.S. average.^31^ Using that index would give a one-surface amalgam cost of $72/1.43 = $50.35 today. It is reasonable then to keep the national average assumption at $54, which is 38% higher than the current California Medicaid payment rate.

The $18 opportunity cost was a U.S. average hourly wage. The 2010 U.S. median and mean hourly wages are reported to be $12.68 and $19.21, respectively.^116^ As equity is the other strongest appeal of CWF, the median wage is more appropriate than the mean wage for representing productivity loss. Substituting the $12.68 for the $18 in equation (4) to obtain an updated average cost per carious surface gives
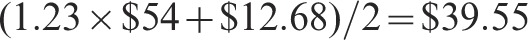
(5)

This value replaces the $72 in equation (1) in Step 2. The final result is that the $19.12 PPPY gross savings in Step 5 changes to
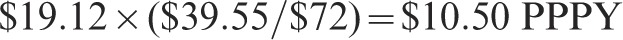
(6)

#### Averted caries — a consistent approach

Calculating averted caries as a product of no-CWF Incidence and CWF Effectiveness is fundamentally unsound. Griffin *et al.*^10^ could have derived a self-consistent averted caries directly from Brunelle and Carlos,^77^ the results from which are summarized in the first six columns in Table 12.

**Table 12:** Summary data from Brunelle and Carlos,^77^ differences between no exposure and lifelong exposure groups, and estimate of averted caries based on the data

Age		Lifelong exposure		No exposure		Years after age 6	Difference in mean DMFS	
	U.S. population	Children examined	Mean DMFS	Children examined	Mean DMFS	Cumulative	Annual
5	2,552,751	227	0.03	229	0.10			
6	3,980,732	705	0.14	645	0.14			
7	3,578,063	764	0.36	780	0.53	1	0.17	0.17
8	3,211,415	782	0.64	757	0.79	2	0.15	0.08
9	3,332,326	766	1.05	811	1.33	3	0.28	0.09
10	3,357,708	802	1.64	710	1.85	4	0.21	0.05
11	3,179,166	716	2.12	756	2.63	5	0.51	0.10
12	3,206,386	649	2.46	687	2.97	6	0.51	0.08
13	3,229,289	616	3.43	613	4.41	7	0.98	0.14
14	3,473,894	590	4.05	600	5.18	8	1.13	0.14
15	3,552,049	504	5.53	559	6.03	9	0.50	0.06
16	3,581,737	529	6.02	551	7.41	10	1.39	0.14
17	3,045,456	515	7.01	535	8.59	11	1.58	0.14
Total	43,280,972	8,165	2.66	8,233	3.24		Average =	0.11

DMFS: decayed, missing, or filled tooth surfaces.

As it was assumed that CWF benefit begins at age 6 years and the caries aversion begins after 1 year of exposure [Inputs (f) and (h) in Table 2], the first annualized data point (difference in DMFS) is at age 7 years with 1 year of exposure. This procedure provides 11 data points, as illustrated in the last three columns in Table 12. Taking the mean of the 11 data points gives the average annual DMFS difference (0.11), which can be used as the averted tooth decay surfaces PPPY.

Thus a self-consistent derivation yields an averted DMFS PPPY of 0.11, not 0.19. Applying this correction to the previous adjustment, the gross savings is further reduced to
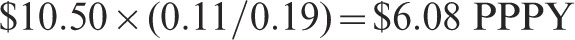
(7)

#### Lack of evidence for adults

Since there is no real evidence that CWF prevents caries in adults, we present hypothetical scenarios; each scenario assumes that the caries aversion rate extends to a given age.

To calculate the estimate for each scenario, Step 4 is modified by summing the weighted costs to the cut-off age. Thus, if CWF is effective to age 19, 29, 39, or 64 years, the national average lifetime cost averted per decayed surface becomes $32.56, $52.98, $74.48, or $100.62, respectively, prior to the corrections. The ratio of each of the lifetime costs to $100.62 is how the gross savings is reduced in each age scenario.

Thus the gross savings of $6.08 PPPY becomes $1.97, $3.20, $4.50, or $6.08 PPPY if the CWF benefit extends to age 19, 29, 39, or 64 years, respectively.

## DISCUSSION

### Corrected net savings

In the previous section, we showed how several defects in the derivation of the $19.12 PPPY estimate of CWF benefit can be corrected. The corrected gross savings estimate is $1.97, $3.20, $4.50, or $6.08, if the CWF benefit extends to age 19, 29, 39, or 64 years, respectively.

As described earlier, the cost estimates of $0.50 for large water systems and $3.17 for small systems^10^ were not based on reality. We used a detailed engineering projection report prepared for a system that has a decade of CWF experience and has characteristics of both large and small systems to obtain a more reasonable estimate of $3 and $10 PPPY, respectively.

The net savings are summarized in Table 13. In short, there is minor savings only if the caries aversion attributed to CWF extends to old ages and only in large systems. Thus minimal correction to several methodological problems eliminates most of the savings. When we include the estimated cost of treatment of dental fluorosis of at least $3.24 PPPY, there are no savings left in any scenario in Table 13.

**Table 13:** Present-day, corrected estimates of net savings ($) per person per year from water fluoridation

			CWF benefit extends to age			
			19	29	39	64
System size	Cost ($)	Benefit ($) ⇒	1.97	3.20	4.50	6.08
Large	3		–1.03	0.20	1.50	3.08
Small	10		–8.03	–6.80	–5.50	–3.92

### Topical effect

There is a question whether any savings for averted caries are real, because the mechanism by which fluoride is thought to help prevent caries is topical. Griffin *et al.*^10^ explained that Assumption (1) in Table 2 was due to the benefit from water fluoridation being primarily “topical and post-eruptive.” The CDC^1^ states that fluoride prevents dental caries predominantly after eruption of the tooth into the mouth, and its actions are primarily topical. Both articles referenced Featherstone,^117^ who stated that the effect of ingested fluoride on caries is minimal.

Current official justification for continuing promotion of CWF is that fluoride in tap water provides teeth with continuous exposure from water, beverages, and foods prepared with tap water, and that a constant low concentration of fluoride is maintained in the dental plaque and saliva all day.^118^ The first point can be left to common sense. The second point contradicts current oral hygiene recommendations concerning plaque and has been refuted concerning saliva. The concentrations of fluoride in ductal saliva, approximately 0.016 ppm in fluoridated areas and 0.006 ppm in nonfluoridated areas, are “not likely to affect cariogenic activity.”^119^

In addition, fluoride, by ingestion or by contact, negatively affects enamel remineralization in individuals with low calcium and magnesium in teeth enamel (usually due to undernutrition).^57^ Hence, CWF may increase caries in people with poor nutritional status.

### Equitable?

That CWF particularly helps the poor at a very low average cost to all has been an integral argument for CWF. We briefly examine the equity aspect.

A major review of the effectiveness of CWF states “There is some evidence [strength of evidence = C] that water fluoridation reduces inequality in dental health across social classes in 5- and 12-year-olds [in England] … The small quantity of studies, differences between these studies, and their low quality rating, suggest caution in interpreting these results.”^71^

In Appendix 5, we point out two studies missing from the review of Truman *et al.*^80^ In the first study Szpunar and Burt^49^ reported that a fluoride concentration of 1.0 or 1.2 mg/l prevented caries, but 0.8 mg/l did not. (The current CWF range is 0.7–1.2 mg/l, and HHS proposed to decrease it to 0.7 mg/l.)^3^ This study chose a predominately white township bordering Detroit, instead of the largely black and long fluoridated Detroit, to represent a fluoridated community. Burt *et al.*^120^ reported that only 0.2% of low-income adults in Detroit in the 14–35 age group (born after CWF started in 1967) were caries free (compared to 55% of children up to age 12+ in the unfluoridated community in Szpunar and Burt).^49^

In the second study, Shiboski *et al.*^121^ found that the prevalence of early childhood caries was not affected by fluoridation status. Among Head Start (low income) children, the most fluoridated ethnic group (Asians, with 69% in fluoridated areas) had the worst tooth decay status. Among non-Head Start children, the most fluoridated ethnic group (Asians, with 81% in fluoridated areas) had tooth decay rates similar to those of white Head Start children, with 12% in fluoridated areas.

Truman *et al.*^80^ stated: “The current burden of poor oral health continues to disproportionately affect communities with large numbers of African Americans, American Indians, Hispanics, the poor, and the disabled of any race or ethnic group.” (See also CDC.)^111^ This was not the case historically. Citing many studies published between 1933 and 1947, Finn^73^ stated that blacks had less caries than whites. On the other hand, recent data indicate that dental fluorosis is more prevalent among blacks and Hispanics,^47^,^111^ suggesting that lack of fluoride is not an explanation for their poorer oral health.

### Conclusion

For decades, the U.S. federal and state governments have promoted CWF to improve dental health of residents at low costs. Yet, in spite of the presumed savings in dental costs to Americans due to widespread use of CWF, employment of dentists is projected to grow by 16% between 2012 and 2022 (vs. 11% for all occupations),^122^ and cosmetic dentistry in the U.S. has grown to be a multi-billion dollar industry.^123^ We have shown that the promise of reduced dental costs was based on flawed analyses. In particular, the primary cost-benefit analysis used to support CWF in the U.S. assumes negligible adverse effects from CWF and omits the costs of treating dental fluorosis, of accidents and overfeeds, of occupational exposures to fluoride, of promoting CWF, and of avoiding fluoridated water. In assessing the benefits, it ignores important large data sets and assumes benefits to adults that are unsupported by data. Thus this analysis, as well as other economic analyses of CWF (Appendix 2), falls short of reasonable expectations for a cost-benefit analysis from a societal perspective. Minimal correction of methodological problems in this primary analysis of CWF gives results showing substantially lower benefits than typically claimed. Accounting for the expense of treating dental fluorosis eliminates any remaining benefit.

## DISCLAIMER STATEMENTS

**Contributors** Both authors have contributed substantially to conception of the study, analysis and interpretation of data, drafting of the article, and critical revision of the article. Both authors have given final approval to the article as submitted.

**Funding** No outside funding was received for this project.

**Conflicts of interest** The authors have no conflicts of interest to declare.

**Ethics approval** Ethical approval was not required.

## Footnotes

i CDC focused on the smallest and largest systems:^9^ for a population <5,000 people, the net savings is $19.12 – $3.17  =  $15.95; for a population >20,000 people, the net savings is $19.12 – $0.50  =  $18.62.

ii These papers are technically cost-benefit analyses,^11^ although the term cost-effectiveness is frequently used to refer to the degree that the value of benefit exceeds cost.

iii While cutting off replacement at age 65 years may appear to underestimate the benefit (cost of fillings), Griffin *et al.* appear to have overestimated by incorrectly or inconsistently applying the factors in the numerator, e.g. the expected lifetime costs of a decayed surface for age groups 55–59 and 60–64 years are both $69.23 = $72/1.04 (Table 3), without the factor (which should be around 0.79) to account for fewer teeth at older ages. (Since Griffin *et al.* did not present these factors, except for the few that appear in the Equation (1) example, we are not able to recalculate and correct the numbers in Table 3.)

iv In a letter to Davis County Health Department and others, the Mayor of Layton, Utah strongly protested that the costs of fluoridation to the citizens of Layton and Davis County were far greater than the costs portrayed by the Department when it “clearly knew better”.^27^

v First 5 Sacramento, the organization that funded the study, may fund the capital update cost with a condition that requires the city to commit to CWF for 20 years, regardless of the city’s future fiscal conditions.^18^ In July 2012, the city accepted a grant from First 5 Sacramento to continue fluoridation until June 30, 2015, even though the grant will provide less than 10% of the system costs over the next 3 years.^30^

vi The service life of fluoridation equipment depends very much on the component and on the service conditions. According to Black and Veatch,^29^ only a few components in indoor setups can expect a service life of 15 years or more, and some components have a service life of only 5–10 years in an outdoor setup.

vii Dean’s classification for very mild, mild, moderate, severe and very severe dental fluorosis: at least two teeth contain mottled surface area covering less than 25%, between 25 and 50%, between 50 and 100%, 100% (with discrete pitting), and 100% (with confluent pitting) of the tooth surface, respectively^51^

viii Infants and young children are most at risk for exposures leading to dental fluorosis.^45^ Children ages 12–15 years when surveyed between 1999 and 2004 were born between 1984 and 1992. Centers for Disease Control and Prevention’s data indicate that 53–60% of the U.S. population between 1984 and 2002 received fluoridated water.^54^

ix 3.6 / 53%  =  6.8% and 3.6 / 60%  =  6%.

x People with high water intake include athletes, outdoor workers, military personnel, and people with medical conditions such as diabetes insipidus or diabetes mellitus.^44^ People with impaired kidney function may have high water intake and might also have reduced urinary excretion of fluoride.^44^

xi DMFS counts the number of decayed (untreated), missing, filled tooth surfaces and DMFT counts teeth instead of surfaces. (An adult without wisdom teeth has 28 permanent teeth and 128 tooth surfaces.) Capital letters refer to permanent teeth and lower case letters (dmfs, dmft) to deciduous teeth.

xii Proponents often appeal to sympathy for young children with high levels of tooth decay to argue for CWF.^82^ However, early childhood caries (ECC) is not prevented by fluoride.^83^,^84^

xiii CDC’s “Healthy People” series “provides science-based, national goals and objectives with 10-year targets designed to guide national health promotion and disease prevention efforts to improve the health of all people in the United States.”^89^ One goal of Healthy People 2000, 2010, and 2020 has been to “increase the proportion of the U.S. population served by community water systems with optimally fluoridated water.”^90^^–^92

xiv “Disparities [in dental health] were noticed across all age groups, among racial/ethnic groups, persons with lower education and income, and by smoking status.”^111^

## Appendix 1: State governments repeating the $1 saves $38 claim

This appendix gives a list of U.S. State governments, other U.S. government agencies, and Canadian sources that repeat the claim that $1 saves $38. Mississippi and Oregon did not repeat CDC’s claim of $1 saves $38. We include them in the list because they repeat the net savings PPPY estimated by Griffin *et al.*^10^ Mississippi provided its own cost estimate ($1 to $2 PPPY), but the estimated net savings are from Griffin *et al.*^10^ All URLs were last accessed on August 13–15, 2013, except Canadian sources, which were last accessed on October 15, 2013. † indicates state legislative records. The list may not be exhaustive.

1. *Alabama*: “For most cities, every $1 invested in fluoridation saves $38 in dental treatment costs.” http://medicaid.alabama.gov/documents/2.0_Newsroom/2.5_Presentations/2.5_Medical_Services_Presentations/2.5_OHCA_Goode_Fluoridation_9-23-10.pdf
2. *Alaska*†: “It is one of the most efficient ways of providing cost effective preventative health care; every $1 spent on fluoridation saves $37 in future dental expenses.” http://www.legis.state.ak.us/basis/get_single_minute.asp?session=24&beg_line=00787&end_line=00956&time=1605&date=20060228&commHES&house=H
3. *Arizona*: “Research shows that every $1 invested in water fluoridation saves $38 in unnecessary costs for dental treatment.” http://directorsblog.health.azdhs.gov/?p=1072
4. *Arkansas*: “Fluoridation saves money. According to the the Centers for Disease Control and Prevention (CDC), every $1.00 spent on fluoridation prevents $38 in dental treatment.” http://www.healthy.arkansas.gov/programsServices/oralhealth/Documents/FactSheetFluoridation.pdf
5. *California*: “Every $1 spent on fluoridation saves $38 in dental treatment costs.” http://www.cdph.ca.gov/programs/Pages/FluoridationInformation.aspx
6. *Colorado*: “Fluoridation is proven to reduce tooth decay over a person’s lifetime, and is a cost-effective prevention strategy, saving $38 for every $1 invested and preventing up to 40 percent of tooth decay.” Select “The State of Health – Full report” on http://www.colorado.gov/cs/Satellite/HealthCareReform/CBON/1251641417543
7. *Connecticut*: “Every dollar spent on fluoridation saves $38 in avoided dental bills.” http://www.ct.gov/dph/lib/dph/drinking_water/pdf/Water_Fluoridation_Fact_Sheet.pdf
8. *Delaware*: “In fact, every $1 invested in fluoridation saves at least $38 in costs for dental treatment.” http://www.dhss.delaware.gov/dhss/pressreleases/2013/cdcfluoridationaward-021313.html
9. *Florida*: “The return on investment is tremendous — with various studies reporting $38–$80 in dental treatment cost savings for each dollar invested in community water fluoridation.” http://www.doh.state.fl.us/family/dental/perspectives.pdf
10. *Georgia*: “Water fluoridation has been shown to reduce dental decay by 20–40% in fluoridated communities, and results in a savings of $38 in future dental expenditures for each $1 invested in fluoridation.” http://www.dph.ga.gov/programs/oral/index.asp
11. *Illinois*: “Studies have shown that for every dollar invested in fluoridation, as much as $38 is saved in dental treatment costs.” http://www3.illinois.gov/PressReleases/PrintPressRelease.cfm?RecNum=2846
12. *Indiana*: “CDC data shows that for every dollar spent on water fluoridation, $38 are saved in reduced costs for dental care.” http://www.in.gov/isdh/23287.htm
13. *Iowa*: “In fact, every $1 invested in water fluoridation saves $38 in dental treatment costs.” http://publications.iowa.gov/6430/1/may_jun2008%5B1%5D.pdf
14. *Kansas*: “For most cities, on average, every $1 spent toward community water fluoridation saves $38 in dental treatment costs.” http://www.kdheks.gov/ohi/download/Burden_of_oral_disease_in_Kansas.pdf
15. *Louisiana*: “Each $1 spent saves $38 in future dental treatment costs.” http://new.dhh.louisiana.gov/assets/oph/Center-EH/operator/04-FlouridePresentation_Exercise.pdf
16. *Maine*: “In fact, for every dollar spent on community water fluoridation up to $42 is saved in treatment costs for tooth decay.” http://www.maine.gov/dhhs/mecdc/population-health/odh/water-fluoridation.shtml
17. *Maryland*: “For most cities, every $1 invested in community water fluoridation saves $38 in dental treatment costs.” http://phpa.dhmh.maryland.gov/oralhealth/docs1/community-water-fluoridation.pdf
18. *Massachusetts*: “In fact, for every dollar spent on community water fluoridation, up to $38 is saved in treatment costs for tooth decay.” http://www.mass.gov/eohhs/docs/dph/com-health/oral-fluoride-community-water-factsheet.pdf
19. *Michigan*: “For most cities, every $1 invested in water fluoridation saves $38 in dental treatment costs.” http://www.michigan.gov/documents/mdch/2012_MOHC_CWF_Tool_Kit_395210_7.pdf
20. *Minnesota*: “Recently published CDC studies have indicated that, for most cities, every $1 invested in water fluoridation saves $38 in dental treatment costs.” http://mn.gov/health-reform/images/WG-PPH-2012-04-Public-Comments-Minnesota-Fluoridation-Plan.pdf
21. *Mississippi*: “In Mississippi, the cost of water fluoridation is usually between one and two dollars per person per year and saves $16 – $19 per person per year in dental treatment costs.” http://www.msdh.state.ms.us/msdhsite/_static/resources/1067.pdf
22. *Missouri*: “For most cities, every $1 invested in water fluoridation saves $38 in dental treatment costs.” http://health.mo.gov/living/families/oralhealth/pdf/oralhealthbrochure.pdf
23. *Nevada*: “It has been estimated that for every one dollar invested in community water fluoridation there is a savings of approximately $38 or more in averted dental treatment costs.” http://health.nv.gov/PDFs/OH/BurdenOfOralDisease2012.pdf
24. *New Jersey^†^*: “An analysis by the CDC has found that, in communities of more than 20,000 people where it costs about 50 cents per person to fluoridate the water, every one dollar invested yields $38 savings in dental treatment costs.” ftp://www.njleg.state.nj.us/20082009/A4000/3709_I1.DOC
25. *New York*: “Every dollar spent on fluoridation on average saves $38 in avoided dental bills.” http://www.health.ny.gov/prevention/dental/fluoridation/cost.htm
26. *North Carolina*: “For every dollar spent on community water fluoridation, approximately $38 is saved in treatment costs for tooth decay.” http://www.ncdhhs.gov/dph/oralhealth/services/fluoride.htm
27. *North Dakota*: “According to the U.S. Centers for Disease Control and Prevention, for every dollar spent on community water fluoridation, up to $38 is saved in treatment costs for tooth decay.” http://www.ndhealth.gov/oralhealth/Publications/2012-2017_Oral_Health_State_Plan.pdf
28. *Ohio*: “Every dollar spent on fluoridation saves more than $40 in dental care.” http://www.odh.ohio.gov/features/odhfeatures/PublicHealthWeek/Friday.aspx
29. *Oklahoma*: “For most cities, every $1 invested in water fluoridation saves $38 in dental treatment costs.” http://www.ok.gov/health/Child_and_Family_Health/Dental_Health_Service/Community_Water_Fluoridation_Program/
30. *Oregon*: “Saves per person per year: $15.95 in small communities; $18.62 in large communities.” http://public.health.oregon.gov/PreventionWellness/oralhealth/Documents/fluoride-program-module1.pdf
31. *Pennsylvania^†^*: “However, for most cities, every $1.00 invested in water fluoridation saves $38.00 in dental treatment costs.” http://www.legis.state.pa.us/WU01/LI/CSM/2009/0/25_X.pdf
32. *Rhode Island*: “For every dollar spent on community water fluoridation, up to $38 is saved in treatment costs for tooth decay.” http://www.health.ri.gov/healthyliving/oralhealth/about/fluoridation/
33. *South Carolina*: “For most cities, every $1 invested in community water fluoridation saves $38 in dental treatment costs.” http://www.scdhec.gov/health/mch/oral/docs/water_fluoridation_flyer.pdf
34. *Tennessee*: “Every dollar spent on fluoridation saves $38 in avoided dental bills.” http://health.tn.gov/oralhealth/communityBenefits.html
35. *Texas*: “A CDC study found that for communities with 20,000+ residents, every $1 invested in community water systems with fluoridation yields $38 in savings from fewer cavities treated.” http://www.dshs.state.tx.us/dental/Oral-Health-in-Texas-2008-Report.doc
36. *Utah*: “… in most communities, every $1 invested in fluoridation saves $38 or more in treatment costs.” http://health.utah.gov/oralhealth/resources/oralHealthReport_2011webFinal.pdf
37. *Vermont*: “For every dollar spent on fluoridation, up to $38 is saved in costs associated with dental care.” http://healthvermont.gov/family/dental/fluoride/
38. *Virginia*: “CDC recommends water fluoridation as a safe, effective, and inexpensive method of preventing decay; every $1 invested in fluoridation saves approximately $38 in costs for dental treatment.” https://www.vdh.virginia.gov/news/PressReleases/2011/110411FlouridationAward.htm
39. *Washington*: “For most communities, every $1 invested in water fluoridation saves $38 in dental treatment costs.” http://www.doh.wa.gov/Portals/1/Documents/Pubs/160-021_Fluoridate_Facts.pdf
40. *West Virginia*: “For every one dollar invested in community water fluoridation, $38 in dental treatment costs are saved.” http://www.wvdhhr.org/mcfh/icah/wv_oral_health_plan_2010.pdf
41. *Wisconsin*: “For most cities, every $1 invested in water fluoridation saves $38 in dental treatment costs.” http://www.dhs.wisconsin.gov/publications/P0/p00457.pdf
42. *Indian Health Service*: “Cost savings — for every $1 spent, $38 saved.” http://www.ihs.gov/doh/clinicmanagement/ohpgdocs/chapter4/community%20water%20fluoridation.doc
43. *National Institute of Dental and Craniofacial Research*: “An economic analysis has determined that in most communities, every $1 invested in fluoridation saves $38 or more in treatment costs.” http://www.nidcr.nih.gov/OralHealth/Topics/Fluoride/StatementWaterFluoridation.htm
44. *Chief Dental Officer of Canada*: “Cooney said the Center for Disease Control estimates every $1 invested in fluoridation saves $38 of dental treatment.” http://www.insidehalton.com/news-story/2895950-fluoride-to-stay/
45. *New Brunswick*: “A study from the Centre for Disease Control in the United States estimated that for every $1 invested in community water fluoridation saves $38 in costs for dental treatment.” http://www2.gnb.ca/content/dam/gnb/Departments/h-s/pdf/en/HealthyEnvironments/FluorideStatement.pdf
46. *Ontario*: “The CDC estimates $38 in avoided costs for dental treatment for every $1 invested in community water fluoridation.” http://www.health.gov.on.ca/en/news/bulletin/2011/hb_20110404_2.aspx
47. *Quebec*: Institut national de santé publique du Québec. “According to the CDC, US$38 could be saved for every dollar invested in fluoridation in a community of 20,000 inhabitants.” http://www.inspq.qc.ca/pdf/publications/705-WaterFluoration.pdf
48. *Saskatchewan*: “Every $1 invested in water fluoridation saves $38 in dental treatment costs.” http://www.health.gov.sk.ca/Default.aspx?DN=7d4df43c-3e21-49cf-9ef2-4b1feca2b4fd
49. *Winnipeg*: “It is also the most cost-effective means of fluoride delivery, with every dollar spent on water fluoridation saving an estimated $38 in treatment costs for tooth decay.” http://www.wrha.mb.ca/wave/2011/11/fluoride-facts.php

## Appendix 2: Other cost-benefit studies

Earlier economic evaluations of CWF have been reviewed by White *et al.*^11^ Mariño *et al.*^124^ summarize a number of studies for caries prevention programs but do not discuss those studies in detail. Griffin and Jones^125^ reviewed Mariño *et al.* Other studies examined dental insurance data and did not find CWF to be associated with lower utilization or costs of dental services.^126^,^127^

In this appendix, we comment on several additional recent CWF cost-benefit studies: Campain *et al.*^128^ assessed the impact of changing dental needs over time on the cost savings from CWF in Australia. O’Connell *et al.*^129^ estimated the cost savings associated with CWF in Colorado and potential savings if the unfluoridated communities were to implement CWF. Wright *et al.*^130^ investigated whether it would be cost-effective to fluoridate water supplies that were not fluoridated in New Zealand. Kroon and van Wyk^131^ examined whether water fluoridation is still a viable option to reduce dental caries in South Africa by addressing concerns about cost and effectiveness. Tchouaket *et al.*^132^ estimate the cost savings in Quebec resulting from CWF; since this is a 2013 paper claiming to use an “innovative approach” we will comment on it separately.

### Costs

As with Griffin *et al.*,^10^ both O’Connell *et al.*^129^ and Kroon and van Wyk^131^ based their cost estimates on Ringelberg *et al.*^14^ Wright *et al.*^130^ hypothetically estimated capital and annual operating costs “by consulting equipment providers and operators of fluoridation systems.” These studies all adopt the assumption of a 15-year replacement schedule except Kroon and Van Wyk,^131^ who are more detailed in the cost aspect and have a separate replacement schedule of 8 years for mechanical and electrical plant. On the other hand, Campain *et al.*^128^ used a simple A$0.27 PPPY but provided no details.

### Estimates of averted caries

O’Connell *et al.*^129^ essentially used the base case from Griffin *et al.*^10^ They used the 25% value for Effectiveness. For Incidence, they used the base case (middle row in Table 6 in the main text) with minor changes: They reduced the 0.77 and 1.09 values by 20.9% for the secular trend, but decided that the 0.43 value for age 45–65 years was too low; instead they used 1.08 and 1.31 for ages 45–64 years and ages 65 years and older, respectively, through consultation with Griffin. The resulting average averted caries is 0.2 DMFS, almost the same value (0.19 DMFS) as in Griffin *et al.*,^10^ but O’Connell *et al.* applied it to all ages from age 5 years.

Campain *et al.*^128^ assumed uniform but changing effectiveness for all ages from age 6 years. They picked a value within the range of numbers reported from a set of references, including several discussed in this article.^77^,^107^,^108^,^133^ Thus they assumed that CWF effectiveness was 50% in the 1970s, 30% in the 1980s, and 25% in the 1990s. For Incidence they constructed a matrix of year versus age range from their literature search and imputed values where information was missing.

Kroon and van Wyk^131^ cited the 15% Effectiveness from Petersen *et al.*,^134^ and also modeled the benefit using 30% and 50%. This Effectiveness is applied to teeth, not surfaces as in the other studies. For Incidence they used local survey data by city. The method, according to Kroon and van Wyk,^135^ is to divide the DMFT survey of, say, 15-year-olds by 15–6 = 9 and assume it is the same for people of all ages, including those age 6 years and less. The authors noted that the mean DMFT for 12-year-old South African children decreased from 1.73 in 1988–1989 to 1.05 in 1999–2002 in this unfluoridated country.

Wright *et al.*^130^ did not try to estimate a value for Effectiveness. For children aged 4–13 years, they compared treatment data for restorations and extractions for both deciduous and permanent teeth to calculate savings on dental fees. They used 1996 Wellington and Canterbury data without supporting the selection, since such data are available for all New Zealand and for all years. For ages 14–34 years, they used a 0.29 averted DFS number from Grembowski *et al.*^107^ (but increased it to 0.59 surfaces for Maori) and assumed no effectiveness after age 34 years.

### Costs of dental treatments

On productivity loss, Campain *et al.*^128^ and O’Connell *et al.*^129^ used approaches similar to Griffin *et al.*^10^ Wright *et al.*^130^ and Kroon and van Wyk^131^ did not include productivity cost. Below, we note the variations in the methods of estimating dental fees in these studies.

Kroon and van Wyk^131^ estimated caries in DMFT and used the average cost of two-surface fillings for the dental charge for each DMFT. Wright *et al.*^130^ used the treatment database from Wellington and Canterbury for children ages 4–13 years and included both deciduous and permanent teeth. For those ages 14–34 years, they calculated the cost of a single-surface filling using an average dentist hourly rate (with inflation) and the 15 minutes time needed to put in the filling. They assumed that fillings are replaced every 8 years.

Campain *et al.*^128^ and O’Connell *et al.*^129^ attempted to include more-surface fillings, composite fillings, and crown or extraction costs. However, the calculations lack transparency, and there are questions as to whether the interaction between extractions and restorations is handled properly in the latter. The most serious problem with the two studies is that they calculated the dental fees plus productivity cost on a *per visit* or *per service* basis, rather than normalizing that cost to a *per surface* basis, because one visit or service may treat more than one surface. By multiplying the estimated averted DMFS by a cost per visit or service rather than a cost per surface, they overestimated the averted costs of dental services. In addition, crowns or extractions are not always due to caries, but may have other causes. Thus these approaches lead to a far worse overestimation than Assumption (6) in Griffin *et al.*’s analysis.^10^

### Tchouaket *et al.*^132^

A paper by Tchouaket *et al.* claims to use an “innovative approach” to assess the economic value of water fluoridation for Quebec, in which only 2.7% of the population is fluoridated.^132^ The presentation lacks critical information and contains fundamental errors. The authors claim that their analysis “adopted a societal perspective that allowed us to track all the costs and effects of the intervention.” However, they did not include or mention the costs of treating dental fluorosis or any of the costs we discussed under “Other costs.” All $ signs in this section are Canadian dollar, C$.

Tchouaket *et al.* produced $1.93, $2.05, or $2.25 PPPY as the costs of CWF, using information from the few fluoridated municipalities in Quebec. Supposedly, the three values correspond to using 3%, 5%, or 8% to amortize the subsidies received by these municipalities over 20 years. They listed several salary rates but provided no other quantitative information, thus readers are not able to repeat any calculations or confirm the numbers.

For CWF benefits, Tchouaket *et al.* did not try to estimate averted caries. Instead, they estimated the yearly costs associated with restorative dental treatments in Quebec to be $532.08, $532.87, or $534.05 PPPY, depending on discount rates. They compared these with the cost values above at various hypothetical values of CWF effectiveness, and claimed that CWF is cost-effective even at 1% effectiveness and that Quebec saves more than $560 million a year at an “expected average effectiveness of 30%.”

It should be noted that the $532–$534 PPPY *restorative* expense exceeds the actual per capita spending on *all* dental services in Canada, which was reported to be $380.83 in 2009 and $399.10 in 2011.^136^,^137^ Tchouaket *et al.* confused untreated tooth decay and dmft/DMFT (decayed, missing, or filled teeth) — only untreated decay (“d” or “D” in dmft/DMFT) requires a restoration service. A filled tooth might need a replacement at some point in the future, but definitely not every year.

The authors calculated the number of teeth restored in a year by multiplying the number of persons who used dental services within the past year, by age group, times the dmft/DMFT index for that age group. First, the average dmft/DMFT values given in the paper are clearly cumulative, not an annual increment. Only a small percentage of these would correspond to untreated decay that requires a restoration service.^xv^ Second, Tchouaket *et al.* apparently failed to recognize that routine dental cleaning and examinations are common in developed countries, thus having used dental services does not equate to having had a tooth restored.

Data in the paper indicate that 25–61% of children (depending on age) were caries free, while 78–91% had visited a dentist in the past year; thus many of the children utilizing dental services had not had any restorations, that year or previously. The 35–44 age group had an average 20 DMFT, and 69% used dental services in the past year. Tchouaket *et al.*’s calculations assumed that each of the 69% (724,000 people) had 20 restorations in 1 year. The correct interpretation of the data is that the average Quebecer 35–44 years old had accumulated 20 DMFT between the age of about 6 and the time of the survey, about 29–38 years, or approximately 0.5–0.7 new DMFT on average per year. This is consistent with the increments of 0.2–0.6 dmft/DMFT for children that can be derived from other information in the paper.

Tchouaket *et al.* summarized fees for treatment of one cavity, including transportation costs and lost wages. A total fee for each of three categories (by type of tooth and age group) was not provided. The text and table in the paper disagree on the calculation of transportation costs, and some information in the summary table is not explained. The text indicates that those age 14 years or older require two separate trips, one for a complete examination and one for the restoration to treat one cavity. However, fees for routine dental exams should not be counted toward costs that can be saved by CWF.

The authors appear to have taken their calculated number of teeth restored for a given age group times the cost per restoration for that age group to obtain the total cost of restorations in one year for that age group. The combined total cost for the three age groups included in the analysis (5–8, 11–14, 35–44; 1.7 million people total) appears to have been averaged over the entire population of Quebec (7.9 million people) to obtain their final average of $532–$534 PPPY. This brings up the question of whether other age groups (9–10, 15–34, and 45+) were assumed to have no restorations. However, averaging (incorrectly) over the entire population rather than over the relevant age groups compensates partly for the great overestimation in the number of restorations per year.

The three values $532.08, $532.87, or $534.05 supposedly differ in the different discount rates (3%, 5% and 8%) used to calculate repeat treatment. Estimating the dental cost for replacement services is not new, but the scant description provided in two sentences does not show what the authors have done or allow readers to understand why the three results are so close.

Tchouaket *et al.* admitted that basing the 2010 economic value on caries prevalence data more than a decade old is a limitation. This is a legitimate concern due to the well known “secular decline” of caries in developed countries.^72^ However, the authors argued that because the percentage of the Canadian population with at least one dental cavity has remained stable at 96%, the average DMFT in Quebec likely has remained the same or even increased. Actually, the 96% figure applies only to dentate adults aged 20–79 years. For children aged 6–11 years and adolescents aged 12–19 years, the corresponding national figures are less than 60%.^136^ Factual error aside, this argument reflects their confusion with the differences between cumulative DMFT and new caries and with properly defined populations.

## Appendix 3: Accidents, overfeeds and damages

A number of accidents, overfeeds, and damages caused by CWF are summarized in Table 14. Additional incidents of acute poisoning have been described elsewhere.^139^,^140^

**Table 14:** Examples of accidents, overfeeds and damages from CWF

Location and date	Description
1. Deltona, FL September, 1994	“A tanker truck cracked open on I-4 near Deltona … and released 4,500 gallons of fluorosilicic acid in one big whoosh.” It was “one of the worst chemical spills in Volusia county’s history.” 2,300 people were evacuated, and more than 50 people were sent to hospitals with complaints of skin and respiratory irritations, including some hours after the spill. Motorists were instructed not to wash off the chemical film with water as that could cause respiratory problems to anyone nearby. EPA officials felt it was “a significant health hazard as far as ground water.” The agency ordered around-the-clock cleanup on I-4 that lasted days.
2. Lowell, AR December, 1996	Beaver Water District fluoridated Fayetteville with fluorosilicic acid and Springdale with sodium fluorosilicate powder prior to 1992. When CWF was resumed in 1996, adding Rogers and Bentonville, the decision was made to use the powder, as fumes from the liquid had severely damaged the injection facility in the past.
3. Malvern, AR March, 1997	A water plant operator at the Kimzey Regional water plant was sprayed by fluorosilicic acid at work. According to his 2012 personal account, he became 100% disabled for almost 14 years and still requires large amounts of pain medicine. He suffered permanent health damage, including losing all his teeth.
4. Charleston, SC August, 2000	A worker accidentally put the wrong chemical in the fluoride tank in the Hanahan Water Treatment Plant. The chemical “reacted; it released a large amount of heat; the fiberglass essentially melted; the gas flowed; it just burst.” This resulted in a 20,000-gallon acidic mess. The total bill for cleanup and repairs was about $250,000.
5. Wakefield, MA August, 2000	An overdose of fluoride seeped into the town water supply. Officials made door-to-door warnings around the pumping station. The public became aware only after a local news station called the town. Authorities said there were no reports of illness; but Linda Collins disagreed, “I was crazy dizzy and I had the runs. I think it was woefully inadequate the way they notified us,” she said. “Because they didn't.”
6. Coos Bay, OR October, 2000	At least 3.5 million gallons of partially treated sewage has spewed into the Coos Bay after 400 gallons of fluorosilicic acid flowed into a sewage treatment plant, killing its bacteria-munching organisms.
7. Fort Wayne, IN February, 2001	About 6,000 gallons of fluorosilicic acid drained from the lower level of the filtration plant into the sewer. The fluoride tank overflowed, and caustic fumes filled the area causing difficulty breathing, chest pains, severe headache and sore eyes in plant workers. Four workers were treated in the hospital.
8. Marlboro, MA October, 2003	A valve malfunction allowed a concentrated level of fluoride to flow into the water system. Workers went door to door to alert nearby customers, flushed water mains, and shut down the plant for some time. Residents and businesses were advised to take extreme care when flushing their pipes, and not to come into contact with the water, which could cause burning, skin irritation, or both.
9. Westminster, MA November, 2005	Emergency crews responded to a chemical spill at the Regional Water Treatment Facility after one of the storage tanks leaked about 750 gallons of fluorosilicic acid. An operator and two colleagues were transported to the hospital.
10. Moncks Corner, SC April, 2006	In the Santee Cooper water treatment plant, a water plant security guard became sick after she walked through a cloud of sodium fluorosilicate. The complaints included having trouble breathing, feeling like something was constantly caught in her throat, and “in the following weeks, Morris’s hair started falling out, she developed a rash on her arms and back, and she continued to be wracked with convulsive fits of coughing.”
11. Nashville, TN March, 2007	Valve malfunctions caused a fluoride overfeed in Harpeth Valley Utilities District. The Incident Event Log showed that an operator noted abnormal measurements starting at 12:40 a.m. 9 March 2007. Plant workers went through the facility shutting off equipment, conducted frequent water samplings and measurements, performed aggressive and continuous flushing, and contacted authorities. They also prepared for door to door public notifications, fielded incoming calls, responded to media requests, and continued sampling throughout the distribution systems until 17 March 2007. They also retained an outside engineering service to review and provide recommendations for the chemical feed systems.
12. Salt Lake City, UT August, 2007	A fluoride tank overflowed at a water treatment plant. Fluoride (1,500 gallons) spilled into a pond, resulting in an advisory to avoid Parleys Creek for several days. Utility workers used sandbags and a makeshift earthen dam to contain the chemical. Four hazmat teams worked to keep the fluoride from flowing beyond a park at the base of Parleys Canyon. Water was released from a reservoir to flush the chemical from the creek.
13. Conway, AR July, 2008	A 42-inch water pipe corroded to the point of failure, due to the fluoride injection port being mounted too close to a chlorine injection port, necessitating the shutdown of a portion of the plant that was completed only in 2005.
14. Chesterfield, MO February, 2009	Approximately 200 gallons of fluoride spilled from a ruptured tanker truck, which was carrying 4,000 gallons of the chemical at Missouri American Water’s central plant. The truck’s driver and two employees from the plant were taken to an area hospital.
15. Anchorage, AK April, 2010	A system malfunction at Fort Richardson Water Treatment Plant caused excess fluoride in the drinking water supply. Officials warned “anyone who lives, works on or visits the two posts in Anchorage not to drink the water … The water also should not be used to brush teeth and wash or cook food. Any ice cubes … should be thrown out.”
16. Asheboro, NC June, 2010	Tank malfunction caused approximately 60 gallons of fluoride to be dispersed into the water system. The news release said: “Residents who consumed a large quantity of water during this period may possibly experience short-term effects such as an upset stomach, vomiting, or diarrhea. The temporary effect from skin contact, such as showering, might include slightly irritated skin.”
17. Rock Island, IL March, 2011	Hazmat crews were called to the Rock Island water treatment plant for a spill of hydrofluorosilicic acid from a tanker truck. As plant employees evacuated, crews began suiting up, working quickly to stop the leak that had begun eating through concrete.
18. England, AR April, 2011	A worker mistakenly poured about 10 to 20 gallons of fluoride into a container holding around 150 gallons of bleach. It created a dangerous gas and led to an evacuation of several businesses near the water treatment plant. The worker and an employee from a nearby business were treated for breathing problems. The county Hazmat team cleaned up the area 3 hours later.
19. Hickory, NC August, 2011	The City transferred $106,713 from capital reserve to maintenance and repair to pay for refurbishing the chemical room and to replace two fluoride tanks. The tanks leaked enough fluoride to degrade the concrete around the containment area and floor.
20. Martinsville, VA February, 2012	The city had to pay $16,450 in penalties after about 1,000 gallons of fluorosilicic acid leaked from a tank at the city water treatment plant. The spill caused the deaths of an estimated 4,445 fish. Officials said that the ground near the spill absorbed quite a bit of the acid, and how much went into the creek was unknown. “Fluorosilicic acid is ‘a very strong acid … with a very corrosive effect on any metals it touches,’ and corrosion caused the pump to fail.”
21. Memphis, TN July, 2012	Fluorosilicic acid tank failure along with containment failure caused approximately 1,500 gallons of the acid to be released onto ground at the public utility. Approximately 1.5 acres were impacted. Workers cordoned off the area and placed berm along the west property line to prevent further runoff. The impacted area was to be excavated and soil properly disposed of.

CWF: community water fluoridation.

Below are the sources, which are mostly media reports, often reproduced in secondary sources, except for the following: Item 3 is a first-person account; Item 11 is an internal log of the water district obtained by request; Item 19 is a city council record; and Item 21 is a report in the National Response Center database. A compilation of other reports in this database up to February 2005 can be found in ActionPA.^141^ All URLs for the sources were last accessed on August 20, 2013, except those in Item 4 that were accessed on April 10, 2014.

1. Deltona, FL. 1994. Spills snarls traffic, lives — The acid closed the road into the night, forced 2,300 from homes and sent 50 to hospitals. Agency orders around-the-clock cleanup on I-4. Orlando Sentinel. http://www.fluoridealert.org/news/fluorosilicic-acid-spill-on-florida-highway/
2. Lowell, AR. 1996. Adding fluoride costly. Carroll County News. http://www.carrollconews.com/story/print/1778486.html and phone conversation with Beaver Water District personnel.
3. Malvern, AR. 1997. The Joe Walls Story by Joe and Jodee Walls, 2012. http://arkansas.securetherepublic.com/www/archiveviewer.php?post_id=1464&post_year=2012
4. Charleston, SC. 2000. Water plant loses fluoride. The Post & Courier. http://nl.newsbank.com/nl-search/we/Archives?p_action=doc&p_docid=111F18B49B49D318&p_docnum=35 and CPW officials treated to plate of headaches. The Post & Courier. http://nl.newsbank.com/nl-search/we/Archives?p_action=doc&p_docid=111F1DB9C2FEDC68&p_docnum=33
5. Wakefield, MA. 2000. Norfolk could teach Wakefield about posting water alerts. Boston Herald. http://www.fluoridefreefairbanks.org/Fluoridation%20Accidents%20Local%20Coverage.html
6. Coos Bay, OR. 2000. Fluoride Spill in Oregon. The Oregonian. http://www.fluoridealert.org/news/fluoride-spill-in-oregon/
7. Fort Wayne, IN. 2001. Fluoride Spill in Fort Wayne, Indiana. The Journal Gazette. http://www.fluoridealert.org/news/fluoride-spill-in-fort-wayne-indiana/
8. Marlboro, MA. 2003. Marlboro water flooded with fluoride — Stuck valve in treatment plant caused toxic release. Worcester Telegram & Gazette. See Item 5 for URL.
9. Westminster, MA. 2005. Two water-treatment workers in hospital after fluorosilicic acid spill. Sentinel & Enterprise. http://www.fluoridealert.org/news/2-water-treatment-workers-in-hospital-after-fluorosilicic-acid-spill/
10. Moncks Corner, SC. 2006. Former water plant guard says chemical cloud made her sick. The Post & Courier. http://www.fluoridealert.org/news/former-water-plant-guard-says-chemical-cloud-made-her-sick/
11. Nashville, TN. 2007. Fluoride overfeed incident event log (March 9–17) and water advisory. Harpeth Valley Utilities District.
12. Salt Lake City, UT. 2007. Fluoride spill taints Parleys Creek. People, dogs warned to avoid stream. The Salt Lake Tribune. http://archive.sltrib.com/printfriendly.php?id=6773022&itype=NGPSID
13. Conway, AR. 2008. Conway Corp. approves funds for fluoridation. The Cabin. http://thecabin.net/stories/071608/loc_0716080001.shtml
14. Chesterfield, MO. 2009. Chesterfield: workers contain spill of approximately 200 gallons of fluoride. St. Louis Post-Dispatch. http://www.fluoridealert.org/news/workers-contain-chemical-spill-of-estimated-200-gallons-of-fluoride-in-chesterfield/
15. Anchorage, AK. 2010. Excess fluoride taints water at Anchorage military bases — Poisonous: High levels can lead to stomach ailments and even death. Anchorage Daily News. http://www.adn.com/2010/04/28/1254268/military-bases-water-supply-overloaded.html
16. Asheboro, NC. 2010. City reports over-release of fluoride. Courier-Tribune. See Item 5 for URL.
17. Rock Island, IL. 2011. Rock Island: hydrofluorosilicic acid spill at Rock Island water-treatment plant. WQAD TV. http://www.fluoridealert.org/news/hazmat-crews-respond-to-chemical-spill-at-rock-island-water-treatment-plant/. Also available in a video at http://www.youtube.com/watch?v=Hvttwsc5lh8.
18. England, AR. 2011. England: mix-up with chemicals at Arkansas water plant causes evacuation; drinking water not threatened. Associated Press reported in The Republic. http://www.fluoridealert.org/news/england-mix-up-with-chemicals-at-arkansas-water-plant-causes-evacuation-drinking-water-not-threatened/
19. Hickory, NC. 2011. City Council Action Agenda. Hickory, NC. http://www.hickorync.gov/egov/documents/1312385507_796722.pdf
20. Martinsville, VA. 2012. Council will pay penalty over spill. Martinsville Bulletin. http://www.martinsvillebulletin.com/article.cfm?ID=32276
21. Memphis, TN. 2012. Spill of 1500 gallons FSA in Memphis, TN. National Response Center Incident Report. Incident Report # 1017173 http://www.nrc.uscg.mil/reports/rwservlet?standard_web+inc_seq=1017173

## Appendix 4: More on CWF effectiveness in adults

We examine two recent articles claiming to show effectiveness of CWF for adults: Slade *et al.*^109^ applied SAS procedures on data from a 2004–2006 Australian survey of adults. Griffin *et al.*^110^ performed a meta-analysis of 20 studies that sought to “examine the effectiveness of self- and professionally applied fluoride and water fluoridation among adults.”

Slade *et al.*^109^ concluded that high lifetime fluoridation exposure was associated with 11% and 10% lower DMFT (or 30% and 21% DFS) among pre-1960 and 1960–1990 birth cohorts. We show that what is attributed to CWF is better explained by differences in age distributions.

Griffin *et al.*^110^ combined CWF with self- and professionally applied fluoride, which are topical treatments and very different from CWF in many respects, e.g. the applications or dosages are controllable; it does not appear reasonable to combine them in a meta-analysis. They “used a random-effects model, which assumes that each study was randomly selected from a hypothetical population of studies,” without discussing the applicability of the model. We focus on the CWF-related studies. These authors concluded that the CWF effectiveness was a 27.2% reduction in caries. We are not able to reproduce this result, which was based on four studies reporting DMFT and one study reporting DMFS; there was no explanation how the different units were handled. As with Slade *et al.*,^109^ Griffin *et al.*^110^ failed to adequately account for different age distributions.

### Slade *et al.*^109^

Thirty dentist-examiners conducted the oral examination in this national survey. For participants aged <45 years, only teeth extracted because of dental caries or periodontitis were counted as missing, but all absent teeth for older people were counted. Fluoridation exposure was determined by residential history, and a value of 0, 0.5, or 1 was assigned if the fluoride concentration at the location was less than 0.3, between 0.3 and 0.7, or greater than 0.7 mg/l, respectively. A value of 0.5 was assigned to all localities in New Zealand, Canada, or the U.S. and 0 to all other foreign localities, without regard to the actual CWF status of the locality.

A significant portion of CWF exposure status was imputed: 3,779 people were considered to have valid exposure data (Complete case), meaning less than 50% of the person’s residential data were missing; the missing years were assumed to be their average observed fluoridation exposure. The exposure status of the remaining 1,726 people with more than 50% missing residential data was imputed by substituting with the status of a random sample from the 3,779 people who belonged to the same geographical stratum and 10-year age group.

Samples were divided into four levels of CWF exposure, i.e. <25% (negligible), 25 to <50%, 50 to <75%, and ≧75% (prolonged) of lifetime. Given the way CWF exposure was determined, the accuracy of this classification is questionable.

Slade *et al.* use unspecified linear regression models to “age-adjust” caries experience and fluoridation exposure. They draw the main conclusion of effectiveness by comparing the “age-adjusted” DMFT/DFS scores of the “prolonged” and “negligible” groups for the cohorts born before 1960 and those born between 1960 and 1990, respectively. The observed DMFT/DFS scores are not provided. The “age-adjusted” DMFT/DFS scores are given by birth cohort and exposure group (Table 15).

**Table 15:** “Age-adjusted” DMFT and DFS from Slade *et al.*^109^

	% of Lifetime exposed to CWF			
	<25%	25–50%	50–75%	≧75%
Pre-1960 birth cohort				
DMFT	21.75	20.90	21.62	19.21
DFS	37.90	35.83	37.00	29.97
1960–1990 birth cohort				
DMFT	8.91	9.53	8.88	7.61
DFS	15.89	18.01	15.65	12.41

CWF: community water fluoridation; DMFT: decayed, missing, or filled teeth.

The scores reported in Table 15 are not consistent with the conclusion that CWF exposure is effective. The scores for the two middle exposure levels were interpreted as “suggested a dose-response relationship.” This is an unreasonable explanation, as an apparent difference in DMFT/DFS is lacking among the first three exposure categories. In addition, exposure levels were defined by cumulative residential status relative to age. For example, a person who lived in places with a fluoride concentration of 1 mg/l for 50 years and 0.25 mg/l for 20 years (treated as 0 mg/l, as 0.25 is less than 0.3) would have been assigned to the 50 to <75% exposure group, which, according to their results, gets no benefit relative to someone living all 70 years in nonfluoridated areas.

There are also questions regarding the validity of their use of linear regression models to “age-adjust.” Calculation from data provided by Slade *et al.* (shown in Table 16) reveals that some cells have few or no people. In particular, the category of ≧75% exposure level is clearly much younger than the other three exposure categories. Given the large difference in DMFT/DFS between the pre-1960 and 1960–1990 birth cohorts (Table 15), and the large difference in age distributions between the first three exposure categories and the fourth category (Table 16), it is not surprising that the ≧75% exposure category would have lower DMFT/DFS scores than the other exposure categories. Hence the differences in caries attributed to CWF between the <25% and ≧75% exposure groups are probably due to inappropriate handling of age distributions.

**Table 16:** Number of people and age distributions in the 2004–2006 Australian National Survey (calculated from information in Slade *et al.*^109^)

	% of lifetime exposed to CWF				
	<25	25–50	50–75	≧75	All categories
15–24	65	10	17	154	246
25–34	91	19	39	268	416
35–44	174	103	187	301	765
45–54	177	143	365	108	792
55–64	212	236	394	0	842
≧65	192	393	134	0	718
Total	910	903	1,135	831	3,779

CWF: community water fluoridation.

There are other unexplained discrepancies. For example, the differences in DMFT or DFS between the <25% and ≧75% exposure groups given in the text are not consistent with the numbers reproduced in Table 15. In particular, the DFS difference in the pre-1960 cohort was said to be 11.10 or 30%, but the numbers indicate 7.93 or 21%.

### Griffin *et al.*^110^

This 2007 article included nine CWF studies (Table 17).^51^,^107^,^133^,^142^^–^147 Few, if any, of the studies can be considered high quality studies appropriate for examining the effects of CWF. Four studies involved concentrations greater than those used for CWF (0.7–1.2 mg/l).^51^,^133^,^143^,^145^ In all, but one study,^146^ the examiners were probably not blind to the location of a subject’s residence. Eight studies were cross-sectional, and the towns compared may have simply differed for reasons having nothing to do with CWF. The one study categorized as “prospective” is in essence a cross-sectional study that compares caries increment over an 18-month period, since no “intervention” was started or changed at the onset of the period.^143^

**Table 17:** Summary of community water fluoridation (CWF) studies included in Griffin *et al.*^110^

Study	Study location	Type of study	High fluoride (>1.2 mg/l)
Eklund *et al.*^51^	USA	Cross-sectional	3.5 mg/l
Grembowski *et al.*^107^	USA	Cross-sectional	
Murray^133^	Great Britain	Cross-sectional	1.5–2.0 mg/l
Englander and Wallace^142^	USA	Cross-sectional	
Hunt *et al.*^143^	USA	“Prospective”	0.7–1.5 mg/l
Wiktorsson *et al.*^144^	Sweden	Cross-sectional	
Stamm *et al.*^145^	Canada	Cross-sectional	1.6 mg/l
Thomas and Kassab^146^	Great Britain	Cross-sectional	
Morgan *et al.*^147^	Australia	Cross-sectional	

Only four of the nine studies were conducted in the U.S. Of these, two were examined earlier in this paper.^51^,^107^ Below we offer a few general remarks, followed by comments on the remaining studies, especially the other two U.S. studies.^142^,^143^

As we discussed earlier, assessment of dental caries in adults is difficult. Wiktorsson *et al.*^144^ described difficulties in judging caries prevalence based on fillings (due to practices such as preventive fillings for discolored fissures on occlusal surfaces) and in defining new caries incidence, since the majority of the primary caries lesions are only enamel lesions, possibly arrested caries in many cases.

In some studies,^133^,^145^,^146^ the reported age distributions suggest that the low fluoride groups were older than the fluoride groups. Griffin *et al.*^110^ do not seem to have considered this difference in the age distributions in their analysis.

In the context of testing the hypothesis that adults benefit by continuing to drink fluoridated waters, the progression of the differences in caries is important. Englander and Wallace,^142^ Murray,^133^ and Stamm *et al.*^145^ each reported narrowing of the differences in mean DMFT between the low fluoride and fluoride groups with increasing ages for lifetime residents. The logical conclusion is that drinking fluoridated water is not helpful beyond a certain age.

Englander and Wallace^142^ examined 896 and 935 adults aged 18–59 years from two Illinois towns, Aurora (1.2 mg/l) and Rockford (0.1 mg/l, referred to as “fluoride deficient”). All subjects were examined by the first author. The caries experience was found to be significantly less in the subjects from Aurora, which was attributed to the different fluoride levels in their drinking water. We offer some observations that disagree with that conclusion.

The differences in mean DMFT presented for the two towns were 5.22, 8.14, 6.62, 5.59, and 5.76 for the 18–19, 20–29, 30–39, 40–49, and 50–59 years old age groups, respectively. The mean years of consuming the respective waters in either city were increased by about 10 years for each additional 10 years age group. However, the difference in mean DMFT decreased for age groups above 29 years (for DMFS, the corresponding differences in the means decreased slightly for ages 30–39 years, but decreased substantially for ages >39 years). If the caries difference is to be attributed to fluoride, are we to conclude that after age 29 years, consuming water with 1.2 mg/l fluoride increases caries?

The study groups from the two cities were said to have similar socioeconomic structures, but there are questions as to how similar the two groups really were. Almost everyone in Aurora (pop. 65,000) and more than half the population of Rockford (pop. 130,000) were contacted. It was found that 2% of those in Aurora over 20 years old were toothless, yet the figure was 14% for Rockford. (Anyone with less than 10 teeth was not invited to participate. The percentages of people contacted who had 1–9 teeth were not given.) A sevenfold difference in the toothless population may indicate an economic difference. Even though the edentulous people were not included in the study, the authors appeared to consider the figures representative, as they tried to adjust the measurements by adding the 2% and 14% toothless figures into the DMFT figures to raise the total percentage difference.

Englander and Wallace^142^ reported their results in DMFT as well as in DMFS. (They wrote that differences in dental caries experience were more striking when evaluated by means of DMF tooth surfaces.) Griffin *et al.*^110^ chose to use the numbers in DMFS. (The ratio of DMFT between the two towns appears to be similar to the ratio of DMFS according to Griffin *et al.*,^110^ but that is because they incorrectly listed the value for the filled component instead of the DMF for Aurora.)

Hunt *et al.*^143^ reported new caries incidence over an 18-month period for 424 adults aged 65 years and older from a “narrowly defined geographical area” in two rural Iowa counties. Of these subjects, 174 were lifelong residents of “fluoride deficient” nonfluoridated communities, and 250 had lived in fluoridated communities (0.7–1.5 mg/l) for various lengths of time. Those who had 5–30 years of residence in fluoridated communities had comparable or worse new caries incidence compared to the lifelong nonfluoridated subjects. The authors thus focused on the remaining 101 persons with more than 30 years of residency in fluoridated communities (40% of the fluoridated sample) to draw the conclusion of effectiveness. Griffin *et al.*^110^ used only the 77 persons with more than 40 years of residency in fluoridated communities (31% of the fluoridated sample). As mentioned above, one would be tempted to conclude from Englander and Wallace^142^ that drinking fluoridated water after age 29 years does not work. Here, we learn that drinking it for less than 30 or 40 years does not work.

Hunt *et al.*^143^ used a cross-sectional approach to compare baseline characteristics of the two groups for those with more than 30 years of residence. After at least 30 years of exposure to fluoridated water, no statistically significant difference in DFS (coronal or root caries) was noticed. In fact, Hamasha *et al.*, describing the same study population, did not even mention fluoride as a possible factor in the long-term caries experience.^148^ Apparently, only in this 18-month period was a difference observed and attributed to fluoride. In a companion paper from the same study, Hunt *et al.* indicated no significant correlation between tooth loss and residence in a nonfluoridated community.^149^

Murray^133^ reported on two towns in Great Britain, one with high fluoride (1.5–2 mg/l) and one with low fluoride (0.2 mg/l). Data were reported in 5-year age groups for general samples and for dentate samples. One interesting finding was that the prevalence of edentulous persons by age was strikingly similar between the two towns, reaching about two-thirds by age 60–65 years. In the author’s terms, the “M” component of the DMFT score was similar in both groups. However, one way to look at this is that fluoride ingestion had little or no effect on the likelihood that a person would have a full set of dentures by age 60–65 years. The difference in mean DMFT was fairly constant in the earlier age groups and significantly narrowed from around age 40 years in the general sample. (The pattern of narrowing difference in DMFT persists after removing edentulous samples.) Murray’s samples differed greatly in their age distributions, with the high fluoride group having approximately twice the fraction (33.2% vs. 16.5%) of people in the 20–24 age group and a substantially smaller fraction (27.1% vs. 44.3%) in the 40–65 age groups. Griffin *et al.*^110^ apparently included these samples without considering that it might not be appropriate.

Wiktorsson *et al.*^144^ compared adults 30–40 years old in Swedish towns with 1 or 0.3 mg/l fluoride. Griffin *et al.*^107^ indicate blinded examiners and unspecified fluoride concentrations, but these descriptions do not fit the actual paper — a single examiner performed examinations in the respective communities and was unlikely to have been blind to subjects’ geography. Persons with non-representative water sources were not examined. After discussing difficulties in scoring caries in adults, Wiktorsson *et al.*^144^ report that the community with “optimal” fluoride had “significantly better” dental health status. However, without summary data for age subgroups, the picture is not entirely clear — the presented scatter plots for filled surfaces and for decayed surfaces (for ages 31–43 years) do not appear to suggest a benefit for continuing consumption of fluoridated water. (This study reports in tooth surfaces only and uses linear regression analysis.)

Stamm *et al.*^145^ deal with 1.6 and 0.2 mg/l fluoride in Canada, and the examiners were not blind to their subjects’ place of residence. The study excluded people with fewer than eight teeth. Griffin *et al.*^110^ included the 17–19 year old group in the total sample from Stamm *et al.*,^140^ although the 15–19 year olds in Murray^133^ were excluded. The low fluoride group included only 1.5% in that age group, versus 6% in the fluoride group. Ages 60+ years made up nearly 18% of the low fluoride group but only 12% of the fluoride group. With respect to progression, the differences in mean DMFT between the high and low fluoride groups decreased with the older age groups, from 5.1 at ages 30–39 years to 1.7 for ages 60+ years.

Thomas and Kassab^146^ included only females up to 32 years old, while they were hospitalized to give birth. A single hospital was used by women from a fluoridated island community (Anglesey) and several nonfluoridated mainland communities in Wales (United Kingdom); lifelong residents were included in the study. Although the authors indicate no significant differences in age group structure of the samples from the two areas, the data show that Anglesey had more in the youngest (<20) age group, 24.1% versus 12.9% and fewer in the oldest (25–29 and 30–32) age groups, 30.0% and 5.9% versus 36.5% and 9.6%. The island of Anglesey was chosen for a demonstration fluoridation study in the 1950s. The experiment was terminated after only 5 years and the whole island was fluoridated based on the mean dmft index for 5-year-old children.

Morgan *et al.*^147^ analyzed data for a group of Royal Australian Navy recruits, mostly males, ages 15–24 years, and with limited education. Griffin *et al.*^110^ used only the results (mean DMFT scores by fluoride history) for 20–24 year olds (208 recruits). Morgan *et al.* indicated only that approximately 20% and 30% of the total sample (1,100 recruits) were considered “fluoridated” and “nonfluoridated” (determined by residential history), respectively, and included in the calculation of the mean DMFT scores. Griffin *et al.* used the percentages to impute the sizes of the “fluoridated” and “nonfluoridated” groups to be 42 and 62, respectively.

## Appendix 5: More on the CWF effectiveness in Truman *et al.*^80^

Despite a reference to 21 papers, Truman *et al.*^80^ based their conclusion of CWF effectiveness on 14 studies grouped into three groups:

- Studies starting or continuing CWF with before and after measurements (Group A-On)
- Studies stopping CWF with before and after measurements (Group A-Off)
- Studies starting or continuing CWF with only post measurements (Group B-On)

They calculated a number of “estimates of effectiveness” using two formulas:

*Group A (before-and-after):* (NoF_pre_ – NoF_post_*)* – (F_pre_ – F_post_)} / NoF_pre_ *Group B (post measurements only):* (F_post_ – NoF_post_) / NoF_post_

The measures were mostly DMFT or dft. See Table 5 for summaries.

The median of the estimates thus calculated for each group was taken to represent the CWF effectiveness for each type of studies, even though median can be sensitive to the studies or estimates included in the set. The results were 29.1% for Group A-On (based on 21 estimates from 7 studies), 50.7% for Group B-On (based on 20 estimates from 7 studies), and 17.9% for Group A-Off (based on 5 estimates from 3 studies). With these numbers the authors concluded that “strong evidence shows that CWF is effective.” Below, we discuss a number of problems.

*Selection of studies*: Of the 14 studies, only one 1956 article is a U.S. study.^93^ The two large-scale, multimillion dollar NIDR surveys were not included, peculiar as this review was to be the basis for setting goals for U.S. public policies. Szpunar and Burt,^49^ a study co-authored by the organizer of the 1989 Michigan Workshop, was not included, nor was Shiboski *et al.*,^121^ a CDC-funded study examining caries in California children by ethnicity and head-start (or low income) status. One would expect the authors of Truman *et al.*^80^ to make particular efforts to include this study because (1) the Pacific Region was the least fluoridated region and hence the main target of a new push for CWF and (2) they posed “What is the effectiveness of CWF in reducing socioeconomic or racial and ethnic disparities in caries burden?” as an important unanswered question.

Among the included studies, Loh^96^ is a review article providing partial information from a 1970 paper; it failed to meet the authors’ stated criteria for inclusion. Another inappropriate inclusion was Hawew *et al.*,^103^ a Libyan study with the goals of demonstrating feasibility of collecting data and recording the caries prevalence in Benghazi (0.8 mg/l). The paper also reports data for a small rural area with 1.8 mg/l fluoride concentration, but the comparison of 0.8 (within the CWF range of 0.7–1.2 mg/l) versus 1.8 mg/l (above the CWF range) is not relevant to demonstrating the effectiveness of CWF.

Some authors of the included studies have published other CWF studies, e.g. Attwood co-authored Downer *et al.*,^150^ which could be a B-On study. Künzel *et al.*^151^ and the many studies cited therein could be in group A-Off. These omissions are particularly surprising since group A-Off has only three studies and five estimates.

*Selection of estimates*: Group A-Off contains three studies and five imputed estimates: 17.9% from Attwood and Blinkhorn,^99^ 29.1% and 31.7% from Kalsbeek *et al.*,^100^ and –1.1% and –42.2% from Künzel and Fischer.^98^ Thus the median is 17.9%.

Kalsbeek *et al.*^100^ reported measurements for 15-year-olds taken in different years. Of the two before-and-after estimates imputed from this study, one was not a before-and-after comparison — the fluoridated town had stopped CWF about 6 or 7 years before the “before” point.

Künzel and Fischer^98^ reported measurements for every age from 6 to 15 years. This was a large-scale multiyear study, and the sizes of each age group are significant. But instead of imputing multiple estimates from selected single-age data, as was done with some other studies, all single-age data were ignored, and only two age-range summaries were used to impute two estimates. Had Truman *et al.*^80^ been consistent in the selection of estimates, the median for Group A-Off would have shifted to a negative value and changed the conclusion to no CWF effectiveness.

In contrast, four estimates were imputed from the much smaller Libyan study^103^ — for each of the two ages reported, the public school data from the rural town were used twice to impute two estimates by comparing them with the public school data and, separately, with the private school data from Benghazi.

*Selection of formula*: Within the limited set of studies and estimates selected, the authors failed to apply their formula consistently. Two estimates for deciduous teeth from Guo *et al.*^76^ were included in group A-On and two for permanent teeth in group B-On. The study clearly belongs to group A-On, as it reported before-and-after measurements for all ages and for both deciduous and permanent teeth. Instead of following their stated method and including the estimates of 300%, 211%, and 208% for the permanent teeth of the three selected age groups, they ignored the before measurements and treated them as if there were only post measurements for permanent teeth. Similarly, Evans *et al.*^97^ reported measurements for 5-year-olds divided into three social classes. Truman *et al.*^80^ included three Group A-On estimates, one for each social class; but the combined total for all social groups was treated as a post-only study and contributed another estimate in Group B-On.

The results from the application of the formula can be misleading. For example, the three positive estimates^99^,^100^ in group A-Off are presented as estimates of how much CWF prevents caries. In fact, the data in these two studies, as well as other studies involving cessation of CWF, showed that there were no increases in caries after stopping fluoridation, aside from possibly a temporary and small increase shortly afterward, which could simply be reflecting the removal of the delayed eruption effect. (Within the 6-year period in Attwood and Blinkhorn,^99^ the mean DMFT decreased by 0.54 in the never-fluoridated town and increased by 0.06 in the town that stopped fluoridation at the midpoint of the period. In the 9-year period that sandwiched the cessation of CWF in Kalsbeek *et al.*,^100^ the DMFT decreased by 3.7 in the never-fluoridated town and increased by 0.4 in the fluoridated town; 8 years later, it further decreased by 5.6 in the former and decreased by 2.3 in the latter town.) Thus the purported positive outcomes were purely an artifact of the formula — the never-fluoridated communities had a dramatic reduction in caries without the help of CWF.
